# Metal-based nanomaterials for cancer phototherapy: current advancements and translational challenges

**DOI:** 10.3389/fphar.2026.1841081

**Published:** 2026-06-04

**Authors:** Hao Su, Xinyu Zhao, Wenchao Xu, Huanlan Sa, Xiaohan Li, Zhibing Liu, Fangling Ning, Mianli Li, Zhijing He

**Affiliations:** Department of Oncology, Binzhou Medical University Hospital, Binzhou, Shandong, China

**Keywords:** cancer, clinical potential, combined therapy, metal-based nanomaterials, phototherapy

## Abstract

Cancer remains a leading global health challenge, with limitations in chemotherapy, radiotherapy, and immunotherapy driving interest in alternative modalities. Phototherapy, comprising photodynamic therapy and photothermal therapy, has shown promising antitumor efficacy, yet conventional photosensitizers and photothermal agents often suffer from poor stability, limited tissue specificity, and suboptimal *in vivo* performance. Metal-based nanomaterials effectively address the above limitations and bottlenecks by providing tunable optical properties, catalytic activity for reactive oxygen species generation, and multifunctional platforms for imaging and combination therapies. In this review, we systematically discuss recent advances in precious metal and non-precious metal nanomaterials for cancer phototherapy, elucidating their diverse mechanisms of action and anticancer efficacy, and highlighting the pivotal role of materials design in performance optimization. We further summarize strategies to design and improve these nanomaterials, with an emphasis on enhancing biocompatibility, tumor targeting, and photothermal conversion efficiency, all of which are critical for clinical translation. We also highlight synergistic therapeutic paradigms that combine metal-based phototherapy with other anticancer modalities, providing representative examples of how such combinations can improve treatment outcomes. Finally, we discuss future directions, particularly the development toward intelligent, multifunctional, and low-toxicity metal-based nanoplatforms through stimulus-responsive designs, functional integration, green synthesis, and artificial intelligence-assisted approaches. Overall, this review aims to provide a solid theoretical and technical reference to accelerate the clinical application of metal-based nanomaterials in cancer phototherapy.

## 1 Introduction

### 1.1 Development background of phototherapy

Cancer remains a formidable challenge to global health, necessitating therapeutic strategies that balance efficacy with safety. While conventional modalities including surgery, chemotherapy, radiotherapy and immunotherapy have significantly advanced oncology, they are often limited by systemic toxicities such as bone marrow suppression, off-target effects, and immune-related adverse events (Citrin, 2017; Szeto and Finley, 2019; Min and Lee, 2022; Letai and De The, 2025). Consequently, there is an urgent demand for localized, highly selective interventions that minimize collateral damage to healthy tissues. Phototherapy (PT) has emerged as a promising approach due to its exceptional spatial control and minimal invasiveness.

The evolution of PT has transitioned from direct light-based ablation to sophisticated, agent-mediated modalities (Bae et al., 2017; Torres et al., 2021; Kaufman et al., 1973; Diwu and Lown, 1994). Following the invention of the laser in 1960, initial clinical applications primarily relied on high-energy thermal ablation of tissues (Maiman, 1960; Goldman, 1966; Alster and Li, 2020; Liu et al., 2023a3; Bai et al., 2024). However, the high power densities required for direct ablation often resulted in significant collateral thermal damage to surrounding healthy structures, thereby compromising the spatial precision and safety profile required for clinical translation (Zhou et al., 2016; Li et al., 2020a). To enhance both safety and efficacy, research has shifted toward drug-assisted phototherapeutic modalities: photodynamic therapy (PDT) and photothermal therapy (PTT) (Wang et al., 2020a; Zhi et al., 2020). PDT utilizes photosensitizers (PSs) to generate reactive oxygen species (ROS) upon irradiation, whereas PTT employs photothermal agents (PTAs) to convert absorbed light into localized heat. Compared to conventional bulk thermal ablation, these agent-assisted approaches offer superior selectivity, reduced invasiveness, and enhanced therapeutic safety.

### 1.2 Application potential of metal-based nanomaterials

The efficacy of both PDT and PTT is fundamentally dependent on exogenous agents, namely, PSs and PTAs. An ideal therapeutic agent should exhibit robust photostability, strong and tunable light absorption within the therapeutic windows, favorable biocompatibility, and preferential accumulation in tumors (Li et al., 2020b; Xie et al., 2020; Xie et al., 2021a; Meng et al., 2022). Although traditional organic small molecules and polymer-based agents offer good biodegradability, they frequently suffer from common drawbacks such as poor photostability (susceptibility to photobleaching), weak near-infrared (NIR) absorption, limited tissue penetration, and sub-optimal pharmacokinetics (Jung et al., 2018; Park et al., 2021; Liu et al., 2019; Jiang et al., 2024a; Wang et al., 2024a).

Metal-based nanomaterials have emerged as a versatile platform to address these challenges (Wang et al., 2022a; Kasparkova et al., 2024; Yu et al., 2025). Attributed to their unique electronic structures, metal-based nanomaterials possess tunable NIR absorption, enhanced photophysical properties (e.g., plasmonic effects), and intrinsic catalytic or enzyme-like activities, which collectively enhance energy conversion efficiency and modulate the tumor microenvironment (Wu et al., 2017; He et al., 2021; Sun et al., 2023; Zhang et al., 2023a; Liu et al., 2025). Furthermore, the physical size and surface chemistry of metal nanoparticles (NPs) can be precisely engineered to improve drug loading and reduce systemic toxicity. For instance, designing ultrasmall particles (e.g., below 5 nm) allows for efficient renal clearance, thereby enhancing their clinical safety profiles (He et al., 2021; Zhang et al., 2023b; Markwalter et al., 2019; Liao et al., 2023). Building on these multifaceted advantages, metal-based nanomaterials have demonstrated significant potential in advancing the performance of both PDT and PTT, as discussed in detail below.

#### 1.2.1 Application potential of metal-based nanomaterials in PDT

Clinical PDT began with early heme porphyrin derivatives, such as hematoporphyrin derivative, which demonstrated activity against superficial tumors but was limited by impurities and prolonged skin retention (Dougherty et al., 1978). Later generations such as 5-aminolevulinic acid (5-ALA) derived prodrugs and other macrocycles improved some pharmacokinetic aspects but tumor specificity remains a limitation for many small molecule PSs (Wang et al., 2024b; Xu et al., 2024). Chlorin e6 (Ce6) is widely used because of high singlet oxygen yield and low dark toxicity, yet its poor aqueous solubility restricts tumor accumulation and therapeutic efficacy (Son et al., 2019). Metal-based nanomaterials have significantly expanded PDT capabilities by serving as PS’ carriers, plasmon-enhanced PS platforms, or photocatalytic agents that promote ROS generation. Metal-based nanomaterials, as excellent carriers, can easily achieve co-delivery of PDT and chemotherapeutic drugs, thereby synergistically killing tumors. For instance, it can increase the sensitivity of tumor cells to cytotoxic drugs by generating ROS, or achieve this goal by altering the tumor’s nutrient supply and immune recognition mechanisms (Huang et al., 2021a). Tumor hypoxia remains a major barrier to PDT (Wen et al., 2022). To alleviate hypoxia, metal-based systems such as manganese containing NPs have been engineered to catalyze the decomposition of endogenous hydrogen peroxide and locally generate oxygen, thereby improving PDT outcomes (Padmanabhan et al., 2016; Duan et al., 2023). The growing diversity of metal-based PS platforms has substantially strengthened the applicability of PDT in oncology.

#### 1.2.2 Application potential of metal-based nanomaterials in PTT

PTT utilizes diverse organic and inorganic PTAs. Organic PTAs possess good biocompatibility yet suffer from low photothermal conversion efficiency (PCE) and poor stability. By contrast, metal-based nanomaterials realize localized surface plasmon resonance (LSPR), which converts photon energy into heat via non-radiative relaxation. This LSPR-based heating enables tumor ablation with lower light dosage than conventional thermal therapy (Liu et al., 2023b; Nanda et al., 2024; Priyadarshi et al., 2025). Gold (Au)- and platinum (Pt)-based nanoparticles have exhibited prominent heat generation and anti-tumor performance under light irradiation (Manikandan et al., 2013). Such nanomaterials feature high tunability via regulating metal composition, particle size and morphology. Precious metals deliver higher PCE than non-precious counterparts, and adjusting their structure can flexibly modulate LSPR absorption peaks. Additionally, rational responsive design grants them tumor microenvironment-targeting capability and optimized biological properties. Overall, metal-based nanomaterials hold great promise for high-efficiency PTT, targeted tumor therapy, synergistic combination treatment and integrated diagnosis and treatment (Elbialy et al., 2019).

### 1.3 Purpose and framework of the review

This review systematically summarizes the latest research progress of metal-based nanomaterials in the field of tumor PT, focusing on their mechanisms of action and antitumor efficacy in PDT and PTT. Compared to previous reviews on tumor treatment related to metal-based nanomaterials, this article categorizes and discusses noble metal-based nanomaterials, non-noble metal-based nanomaterials, metal-organic frameworks (MOFs), and rare earth nanomaterials, comprehensively summarizing the current research status of various metal-based nanomaterials used in tumor PT and PT combined with other therapies (Wang et al., 2025a; Wang et al., 2025b). At the same time, it analyzes their biological safety and clinical translation potential, and further outlines the key research directions and development paths for this field in the future, aiming to provide theoretical basis and practical reference for the application of metal-based nanomaterials in cancer treatment.

## 2 Basic principles of PT

### 2.1 Principles of PDT

**FIGURE 1:**
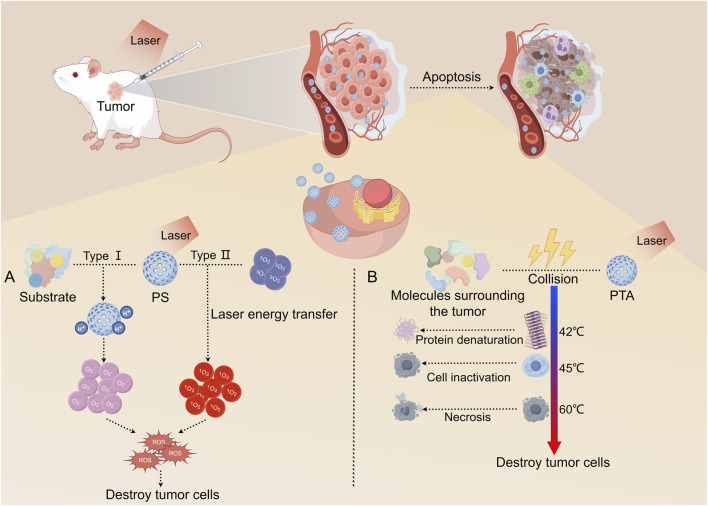
Intratumoral injection of PSs/PTAs into tumor-bearing mice induces apoptosis in tumor cells under laser irradiation at a specific wavelength. **(A)** Mechanism of action of PDT. **(B)** Mechanism of action of PTT.

PDT is an antitumor treatment that employs light of specific wavelengths to activate PSs. Upon entering an excited state, PSs transfer energy or electrons to surrounding oxygen molecules, thereby generating ROS that induce tumor cell apoptosis. The core mechanism of PDT depends on the synergistic interaction among PSs, light sources, and oxygen, ultimately leading to ROS generation. During PDT, photons of specific wavelengths are absorbed by PSs, causing them to transition from the ground state to an excited state. Subsequently, through non-radiative transitions, PSs enter a metastable triplet state. In this triplet state, PSs undergo photosensitized reactions with molecular oxygen or other substrates, resulting in ROS (Pham et al., 2021; Yu et al., 2022). Photosensitized reactions can be categorized into Type I and Type II: Type I reactions are relatively less dependent on oxygen and can proceed partially under hypoxic conditions, where excited-state PSs undergo electron or hydrogen atom transfer with substrates to generate reactive intermediates such as free radicals, e.g., superoxide anion radical (·O_2_^−^) (Dolmans et al., 2003; Kolarikova et al., 2023; Jiang et al., 2024b; Wang et al., 2025c); Type II reactions involve excited-state PSs directly transferring energy to adjacent triplet molecular oxygen (^3^O_2_), exciting it to singlet oxygen (^1^O_2_). ^1^O_2_ is a key form of ROS (Dougherty et al., 1998; Dolmans et al., 2003; Li et al., 2025a). The mechanism of PDT is illustrated in Figure 1A.

Both types of photosensitive responses ultimately lead to the generation of ROS. ROS induce apoptosis or necrosis in tumor cells by oxidatively damaging critical structures such as cell membranes, mitochondria, and nucleic acids. Therefore, the antitumor efficacy of PDT depends on the precise regulation of PSs spectral characteristics, light source parameters, and tissue oxygen concentration.

Second, the wavelength of light plays a decisive role in the biological effects of PDT. Ultraviolet (UV) photons have higher energy, enabling them to directly induce photochemical reactions such as DNA damage and protein photolysis. However, macromolecules like proteins and nucleic acids in biological tissues strongly absorb and scatter UV radiation, limiting its tissue penetration. Consequently, UV light primarily affects superficial tissues (Hou et al., 2015; Tang et al., 2018; Zeng et al., 2024a). In contrast, NIR light resides within the “optical window” of biological tissues and can be further subdivided into two bands: NIR-I (750–900 nm) and NIR-II (1000–1350 nm) (Wan et al., 2023; Kong et al., 2025). Light in this spectral band exhibits low absorption coefficients and weak scattering, allowing for deeper tissue penetration. Even under low-energy irradiation conditions, NIR can be partially converted into thermal energy through non-radiative relaxation processes, producing controllable thermal effects that modulate cellular functions or promote tissue repair (Costa et al., 2019).

Beyond wavelength, the efficacy of PDT is constrained by multiple factors, including ROS lifetime, tumor hypoxia, and the type of PSs (Shen et al., 2021; Alhoussein et al., 2025). Among these, the selection of PSs is particularly critical. Traditional PSs, such as indocyanine green (ICG), porphyrin-lipid conjugates, graphene oxide, and carbon nanotubes, often suffer from low solubility and insufficient uptake by tumor cells. To overcome these limitations, metal-based nanomaterials have been introduced into PDT research. By leveraging their superior optical properties and drug delivery capabilities, these systems have significantly enhanced the bioavailability and therapeutic efficacy of PSs (Hong et al., 2016; Younis et al., 2021; Tan et al., 2025).

### 2.2 Principles of PTT

PTT has demonstrated therapeutic efficacy in various tumor animal models, including bone metastasis, lung metastasis, and lymph node metastasis (Zou et al., 2016; Zhi et al., 2020). Furthermore, combining PTT with existing tumor therapies significantly enhances antitumor effects, such as integrating ultrasound-assisted hyperthermia with chemotherapy drugs like doxorubicin (DOX) and paclitaxel (PTX) (Luo et al., 2021; Sun et al., 2022; Shi et al., 2023).

In summary, the therapeutic principle of PTT is based on heat generated by laser-induced PTAs colliding with molecules surrounding the tumor and subsequently returning to their ground state. This localized heating induces degeneration, inactivation, oxidative stress, and necrosis in tumor cells or tissue (Li et al., 2020a; Wang et al., 2020a; Ren et al., 2022; Dong et al., 2024). At 42 °C, protein denaturation and temporary cell inactivation occur. At 43 °C–45 °C, prolonged inactivation induces oxidative stress in cells. When temperatures rise to 45 °C–60 °C, cells undergo immediate necrosis (Huang et al., 2021b; Chi et al., 2025). Notably, tumor tissues exhibit lower heat tolerance, increased acidity, and greater hypoxia compared to normal tissues, making them more susceptible to thermal effects. This characteristic indirectly enhances the selectivity of PTT (Wang et al., 2023; Jia et al., 2025). The mechanism of action of PTT is illustrated in Figure 1B.

Based on the mechanism of thermal damage, two PTT treatment approaches currently exist.

The first involves continuously exposing tumor tissue to temperatures exceeding 45 °C for several min, killing cells through thermal ablation (Liu et al., 2021). The second method heats tumor tissue to 42 °C–43 °C and maintains this temperature, simultaneously damaging tumor tissue and enhancing its vascular permeability. This facilitates better accumulation of PTAs within the tumor tissue (Huang et al., 2021b; Zhang et al., 2024). Building upon the therapeutic principles of PTT, various PTAs have been developed, including organic dye molecules, organic NPs, precious metal materials, carbon-based materials, and other inorganic materials. These agents further enhance the antitumor efficacy of PTT (Jung et al., 2018; Xie et al., 2020).

## 3 Applications of metal-based nanomaterials in PT

**FIGURE 2:**
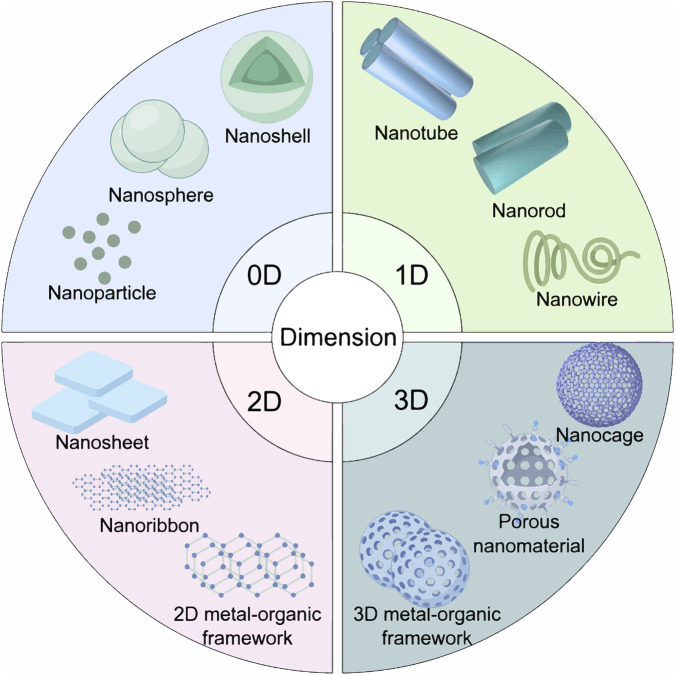
Classification of metal-based nanomaterials at the spatial scale.

Over the past few decades, nanoscale anticancer drugs have been widely used in cancer treatment fields such as radiotherapy, immunotherapy, and chemotherapy due to their excellent targeting properties, minimal side effects, high bioavailability, and potent biological activity. In recent years, they have also seen extensive development in the field of PT. The metal-based nanomaterials discussed in this paper primarily include the following types: metal micelles, metal-based NPs, metal-based nanosheets (NSs), metal-phenolic networks (MPNs), rare-earth-doped upconversion NPs (UCNPs), and MOFs (Ngo et al., 2020). Additionally, from a spatial scale perspective, nanomaterials can be categorized as follows: zero-dimensional (0D), which primarily includes NPs, nanodots (NDs), nanoclusters (NCs), nanospheres (NSPs), and nanoshells, such as those based on Au, silver (Ag), and copper (Cu). One-dimensional (1D): Primarily includes nanorods (NRs), nanowires, nanofibers, nanotubes (NTs), etc. Examples include Au NRs, Cu NRs, and Ag nanowires. Two-dimensional (2D): Primarily encompasses NSs, films, nanoribbons, nanolayered structures, etc. Examples include metallic nanolayers, two-dimensional metal sulfide NS, and 2D MOFs. Three-dimensional (3D) structures, primarily including nanocages, nanoframes, and porous nanomaterials, with 3D MOFs being one example (Baker et al., 2013; Zhang et al., 2025). A detailed classification diagram is shown in Figure 2.

Furthermore, in the field of PT, metal-based nanomaterials can function both as carriers for PSs/PTAs and as PSs/PTAs themselves. Depending on the type of metal, these nanomaterials can be classified into precious metal-based and non-precious metal-based nanomaterials.

### 3.1 Application of metal-based nanomaterials in PDT

#### 3.1.1 Metal-based nanomaterials as PSs’ carriers

Metal-based nanomaterials, used as carriers for PSs, have demonstrated significant advantages in tumor PDT. These nanomaterials can markedly enhance the water solubility and stability of PSs while exhibiting targeted delivery capabilities (Cheng et al., 2022). They not only promote oxygen generation but also directly produce ^1^O_2_, effectively addressing hypoxia in tumor tissues and reducing the oxygen dependency of conventional PDT. Currently, carriers such as metal nanomaterials, metal oxide nanomaterials, metal sulfide nanomaterials, MOFs, and UCNPs have been successfully applied in this field (Wang et al., 2021).

Based on the metal matrix material used, these carriers can be classified into precious metal-based nanomaterials carriers and non-precious metal-based nanomaterials carriers. For instance, Yin et al. (Yin et al., 2020) prepared a nano-dumbbell structure material loaded with ICG using Au NRs as the substrate, Au@mSiO_2_-ICG nano-dumbbells. The presence of Au NRs ensured excellent photostability of ICG and enhanced the efficiency of triplet energy transfer from ICG to oxygen. The Au@mSiO_2_-ICG nano-dumbbells exhibited outstanding uniformity and water dispersibility, with strong plasmonic absorption near 800 nm. Compared to using Au NRs or ICG alone, these nano-dumbbells demonstrated significant potential for enhanced ROS production and photodynamic effects. The modified Au nanomaterials could enhance ICG uptake in tumor cells through passive and active targeting mechanisms to improve PDT efficacy.

**FIGURE 3:**
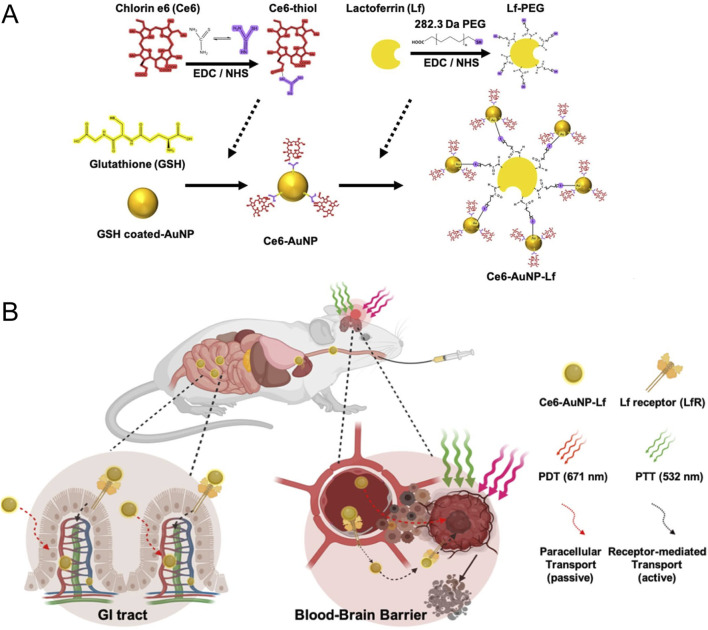
**(A)** Synthetic procedure of Ce6-AuNP-Lf by thiolation of Ce6 and PEGylation of Lf. **(B)** The mechanism of action of Ce6-AuNP-Lf (Kim et al., 2022).

Another study involved the development of Ce6-AuNP-Lf by Kim et al. (Kim et al., 2022) for treating glioblastoma multiforme (GBM). This approach utilized glutathione (GSH)-coated Au NPs to deliver Ce6, and polyethylene glycol-modified lactoferrin (Lf-PEG) was conjugated to the Au NPs surface. The flowchart for synthesizing Ce6-AuNP-Lf is shown in Figure 3A. Lactoferrin (Lf) enabled oral administration of Ce6-AuNP-Lf. Lf facilitated interaction with lactoferrin receptors (LfR) in the gut and blood-brain barrier, enabling efficient passage. Compared to unmodified Ce6, Ce6-AuNP-Lf demonstrated a 1.6-fold increase in ROS generation efficiency. The mechanism of action of Ce6-AuNP-Lf is shown in Figure 3B.

Non-precious metal-based nanomaterials have emerged as ideal carriers for PSs due to their low cost, abundant availability, and inherent catalytic or redox activity. Tumor-targeting strategies utilize physical forces, such as external magnetic fields, to enhance drug accumulation within tumor tissues. Iron (Fe) is one of the most common and strongly magnetic metals (Zhang et al., 2023a). Magnetic targeting differs from active tumor targeting, which relies on ligand-receptor binding, because it is not limited by specific receptor expression patterns. For example, Li et al. (Li et al., 2013) synthesized Fe-based PEG-modified Fe oxide (Fe_2_O_3_) NCs (IONC) loaded with Ce6, termed IONC-PEG-Ce6. Following injection of IONC-PEG-Ce6 into mice, *in vivo* imaging under magnetic field guidance revealed its accumulation within tumor tissues. Quantitative analysis of fluorescence imaging data revealed that magnetic targeting enhanced Ce6 fluorescence intensity within the tumor region by 8.9-fold. Furthermore, IONC-PEG-Ce6 demonstrated excellent *in vivo* safety due to IONC’s good biodegradability and negligible toxicity. Collectively, these findings indicated promising prospects for IONC-PEG-Ce6 in the field of PDT. This example clearly demonstrated the unique advantages of non-precious metal-based nanomaterials combined with physical targeting strategies in PDT. Not only leveraged the properties of non-precious metals to reduce costs and enhance safety but also overcame the traditional reliance on receptor-mediated targeting through magnetic guidance. This approach offerred a novel, efficient, safe, and highly versatile method for drug loading and targeting based on physical principles in tumor PDT. Consequently, it was expected to facilitate the widespread application of non-precious metal-based nanomaterials in tumor PDT.

Additionally, rare-earth metal-synthesized UCNPs as delivery carriers for PSs have garnered significant attention. Researchers have discovered that UCNPs can also achieve a NIR absorption window, enabling their application in PDT targeting deep tissues. For instance, Buchner et al. (Buchner et al., 2019) synthesized RB-lysine-UCNPs using the rare earth metals gadolinium (Gd) and ytterbium (Yb), with rose bengal (RB) as the PS. RB-lysine-UCNPs absorbed NIR light, and the UCNPs converted this NIR energy into UV and visible light, enabling RB to exert PDT effects. During this process, the Gd and Yb in the UCNPs minimize energy loss during light conversion. As these nanomaterials continue to mature, metal-based nanomaterials PSs’ carriers hold promise for playing an increasingly significant role in tumor treatment through PDT.

#### 3.1.2 Metal-based nanomaterials as PSs

Precious metal-based nanomaterials have emerged as exemplars of highly efficient PSs due to their tunable LSPR and outstanding dual functionality in both PS and PTA. For instance, Yu et al. (Yu et al., 2016) employed a novel synthetic approach to synthesize atomically precise BSA-Ag_13_ NCs based on Ag, using bovine serum albumin (BSA) as a reference. BSA endowed BSA-Ag_13_ NCs with favorable cellular uptake and biocompatibility. When compared to RB, BSA-Ag_13_ NCs exhibited a 1.26-fold higher quantum yield for ^1^O_2_, surpassing most commercially available PSs. Zhang et al. (Zhang et al., 2020) fabricated 2D Au NSs via ion-beam epitaxy. These NSs exhibited uniform thickness distribution between 2 nm and 8.5 nm and demonstrated extremely low cytotoxicity. Upon NIR irradiation, Au NSs stably generated ^1^O_2_ via LSPR, killing cancer cells within 5 min of exposure while generating minimal heat. In summary, precious metal-based nanomaterials outperform conventional PSs in ^1^O_2_ generation efficiency while mitigating issues such as poor cellular uptake, high cytotoxicity, and low biocompatibility. They demonstrate significant potential as PSs in the field of PDT.

**FIGURE 4:**
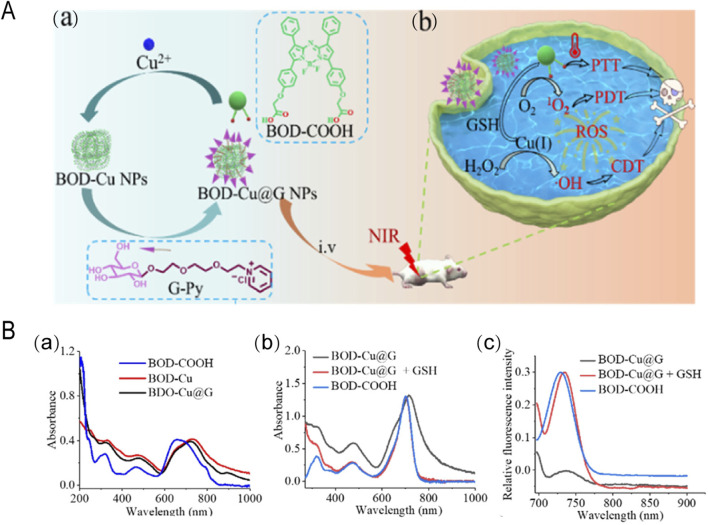
**(A)** (a) The construction of BOD-Cu@G NPs. (b) The mode of action in tumor treatment. **(B)** Characterization of BOD-Cu@G NPs. (a) UV-vis spectra of BOD-COOH, BOD-Cu NPs, and BOD-Cu@G NPs in water. (b) UV-vis spectra and (c) fluorescence spectrum of BOD-COOH, BOD-Cu@G NPs, and BOD-Cu@G NPs + GSH in DMSO (Jin et al., 2024).

Non-precious metal-based nanomaterials offer significant advantages for PDT in deep tissues or challenging environments due to their cost-effectiveness, broad spectral response, and activation via high-energy radiation. Addressing the ROS generation challenge posed by tumor hypoxia mentioned earlier, Jin et al. (Jin et al., 2024) developed glycated nanometallic PS BOD-Cu@G NPs based on Cu, whose construction, mode of action and characteristics are shown in Figures 4A,B. This formulation utilized carboxylic acid-substituted aza-BODIPY (BOD-COOH) as a ligand with both PDT and PTT activity. BOD-COOH targeted tumor cells via the aerobic glycolysis effect (Warburg effect) through its surface glucose derivative (G-Py). Under light irradiation, the dual-therapy ligand BOD-COOH generated ^1^O_2_ and localized hyperthermia, enabling both PDT and PTT. Additionally, the Cu coordination node could increase ROS levels in tumor tissues by depleting reduced GSH within tumor cells. *In vitro* cellular assays revealed that BOD-Cu@G NPs exhibited potent inhibitory effects against multiple tumor cell lines. *In vivo* antitumor evaluations further demonstrated that BOD-Cu@G NPs significantly enhanced PDT efficacy compared to BOD-COOH. The research findings of Jin et al. clearly demonstrated the unique advantages and potential of non-precious metal-based nanomaterials in addressing complex challenges in tumor treatment. Through innovative design, the properties of non-precious metal Cu were harnessed not only to overcome the limitations imposed by the hypoxic tumor microenvironment to ROS generation, but also to achieve precise targeting via glycosylation. Additionally, the integration of multiple therapeutic mechanisms enhanced treatment efficacy. This study provided an effective strategy for resolving key challenges in tumor PDT, but also highlighted the significant value of non-precious metal-based nanomaterials in optimizing and expanding the applications of PDT. These advancements may drove further development and clinical translation of PDT for tumors in deep or specialized environments.

MOFs represent a novel class of organic-inorganic hybrid porous nanocarriers that have garnered significant attention in the biomedical field. Their development has further enhanced the loading capacity and stability of PSs. Moreover, due to the high diffusion rates of oxygen and ROS within porous materials, MOFs hold great promise for achieving more efficient PDT (Gao et al., 2024). Liu et al. (Liu B. et al., 2023) assembled DOX-loaded imidazolidine framework (ZIF-8) onto the surface of zirconium (Zr)-based porphyrin MOF, designing PCN@D/ZIF. In this system, ZIF-8 degraded within tumor tissues to facilitate DOX release, while the porphyrin MOF generated abundant ROS through photon capture, significantly enhancing the efficacy of combined chemotherapy and PDT. Hao et al. (Zhao et al., 2025) developed a multifunctional single-laser-triggered nanoplatform (Fe-THBQ/SR) by loading the stimulator of interferon (IFN) genes (STING) agonist (SR-717) into Fe-tetrahydroxy-1,4-benzoquinone (Fe-THBQ) NMOF. Fe-THBQ, a PS effective in the NIR-II window, generated abundant ROS upon 1064 nm laser irradiation. Furthermore, Fe-THBQ/SR released SR-717 upon GSH stimulation to promote STING activation. This process contributed to tumor vasculature normalization and alleviation of hypoxia in the tumor microenvironment, thereby enhancing the efficacy of PDT.

Zinc (Zn)- and Titanium (Ti)-based nanomaterials hold potential as PSs due to their low toxicity, excellent biocompatibility, and photocatalytic properties. However, their wide bandgap necessitates the use of higher-energy UV light with shorter wavelengths compared to other metal-based nanomaterials (Fatima et al., 2022; Sargazi et al., 2022). Yang et al. (Yang et al., 2017) discovered that this issue could be addressed through Au NCs modification. Consequently, they synthesized black anatase-type TiO_2-x_ NT with immobilized Au NCs, Au_25_/B-TiO_2-x_ NTs. The NTs exhibited a thickness of approximately 2 nm and demonstrated outstanding photo-responsive properties. Under Au NCs modification, the optical response range of TiO_2_ was successfully extended to the visible and even NIR regions, where high tissue penetration depth was achieved. When irradiated with NIR light, the NTs significantly amplified ROS generation due to photocatalytic synergistic effects, thereby enhancing the therapeutic efficacy of PDT. This not only offerred innovative solutions for overcoming the application challenges posed by the wide bandgap of Zn- and Ti-based nanomaterials, but also further demonstrated the crucial role of strategic material compositing and modification in optimizing the PDT performance of metal-based nanomaterials. These advancements were expected to promote the broader application of Zn- and Ti-based nanomaterials in tumor PDT.

In summary, compared to precious metals such as Au and Ag, non-precious metals offer abundant availability, lower costs, and exhibit good biocompatibility following appropriate surface modification. They present relatively lower long-term toxicity risks to normal tissues while maintaining the ability to generate ROS, making them promising next-generation PSs for PDT against malignant tumors. The applications of both precious metal-based and non-precious metal-based nanomaterials in PDT are detailed in Table 1.

**TABLE 1:** Application of metal-based nanomaterials in PDT.

Metal type	Name	PS	Light source	*In vitro* anti-cancer effect	*In vivo* anti-cancer effect	Biosafety	References
Au	Au@mSiO_2_-ICG NDs	ICG	*In vitro*: 800 nm	At a concentration of 65.5 μm mL^-1^ of Au@mSiO_2_-ICG NDs, the mortality rate of CNE2 cells reached 99.2%	N/A	N/A	Yin et al. (2020)
​	Ce6-AuNP-Lf	Ce6	*In vitro* and *in vivo*: 532 nm	At a concentration of 5 μM Au NPs and 2.5 μM Ce6, the apoptotic cell population accounted for 45.9% of the total	Following oral or intravenous administration of Ce6-AuNP-Lf at a dose of 60 mg kg^-1^ to mice, the proportion of GBM in mouse brain tissue decreased to 11.8% ± 9.8% and 9.5% ± 10.3%, respectively	The group to which PDT + PTT was applied after oral or intravenous injection administration maintained the body weight from the beginning of treatment	Kim et al. (2022)
​	2D Au NSs	2D Au NSs	*In vitro*: 808 nm	Without introducing additional PSs, 17 μg of 5 nm thick 2D Au NS exhibited a 75% 4T1 cell killing rate	N/A	N/A	Zhang et al. (2020)
Fe	IONC-PEG-Ce6	Ce6	*In vitro* and *in vivo*: 704 nm	As the concentration of IONC-PEG-Ce6 increased from 0.08125 μg mL^-1^ –2.6 μg mL^-1^, the viability of 4T1 cells was almost completely eliminated	Tumor growth in the same group of mice exposed to the magnetic field was significantly suppressed, showing minimal tumor volume increase over the 16-day course following PDT treatment	The mice exhibited normal behavior after being injected with IONC-PEG-Ce6, and H&E staining of the major organs indicated that IONC-PEG-Ce6 did not cause significant toxic side effects in the treated animals	Li et al. (2013)
​	Fe-THBQ/SR	Fe-THBQ	*In vitro* and *in vivo*: 1064 nm	As the concentration of Fe-THBQ/SR increased from 0 μg mL^-1^ –200 μg mL^-1^, the viability of 4T1 and B16-F10 cells was almost completely eliminated	Tumor growth was inhibited in 59.4% of 4T1-bearing mice, while tumor inhibition reached 100.0% in B16-F10 tumor-bearing mice	Over the 14 days observation period, mice exhibited negligible changes in body weight across all treatments. No obvious changes in all indicators of blood from mice after 30 days of intravenous injection of Fe-THBQ/SR reconfirmed the excellent biosafety of the treatment	Zhao et al. (2025)
Zr	PCN@D/ZIF	Porphyrinic MOFs	*In vitro* and *in vivo*: 640 nm	As the concentration of PCN@D/ZIF increased from 12.5 μg mL^-1^ –200 μg mL^-1^,the mortality rate of 4T1 cells increased to 80.1%	Following administration of a 21 mg kg^-1^ dose of PCN@D/ZIF to mice, tumor inhibition reached 66.5% after 18 days of treatment	The H&E staining images of the main organs after different treatments were carried out and demonstrated no obvious tissue abnormalities	Liu et al. (2023c)
Gd, Yb	RB-lysine-UCNPs	RB	*In vitro*: 980 nm	At a concentration of 15 μg mL^-1^ of RB-lysine-UCNPs, approximately 67% of SK-BR-3 cells were killed	N/A	N/A	Buchner et al. (2019)
Ag	BSA-Ag_13_ NCs	BSA-Ag_13_ NCs	*In vitro*: 150 mW white light	As the concentration of BSA-Ag_13_ NCs increased from 50 × 10^-6^_M_ to 500 × 10^-6^_M_, the survival rate of MCF-7 cells decreased to 28% ± 2%	N/A	N/A	Yu et al. (2016)
Cu	BOD-Cu@G NPs	BOD-Cu@G NPs	*In vitro* and *in vivo*: 685 nm	As the concentration of BOD-Cu@G NPs increased from 0 μg mL^-1^ –10 μg mL^-1^, the relative mortality rate of A549 cells reached 80.0%	After 20 days of cultivation, BOD-Cu@G NPs demonstrated significant tumor-suppressing effects in A549, HeLa, HepG2, and MCF-7 tumor-bearing mice	The tissue sections of the major organs of BOD-COOH group and BOD-Cu@G NPs group had no noticeable histopathological lesions	Jin et al. (2024)
Ti	Au_25_/B-TiO_2-x_ NTs	Au_25_/B-TiO_2-x_ NTs	*In vitro*: 650 nm	As the concentration of Au_25_/B-TiO_2-x_ NTs increased from 7.8 μg mL^-1^ –500 μg mL^-1^, nearly all HeLa cells were killed	After 14 days of cultivation, the tumor volume and weight in the Au_25_/B-TiO_2-x_ NTs group were significantly smaller than those in the other groups	The complete blood count assessment and serum biochemistry assay further indicate barely potential toxicity of Au_25_/B-TiO_2-x_ NTs after 14 days treatment	Yang et al. (2017)

### 3.2 Applications of metal-based nanomaterials in PTT

#### 3.2.1 Metal-based nanomaterials as PTAs’ carriers

**FIGURE 5:**
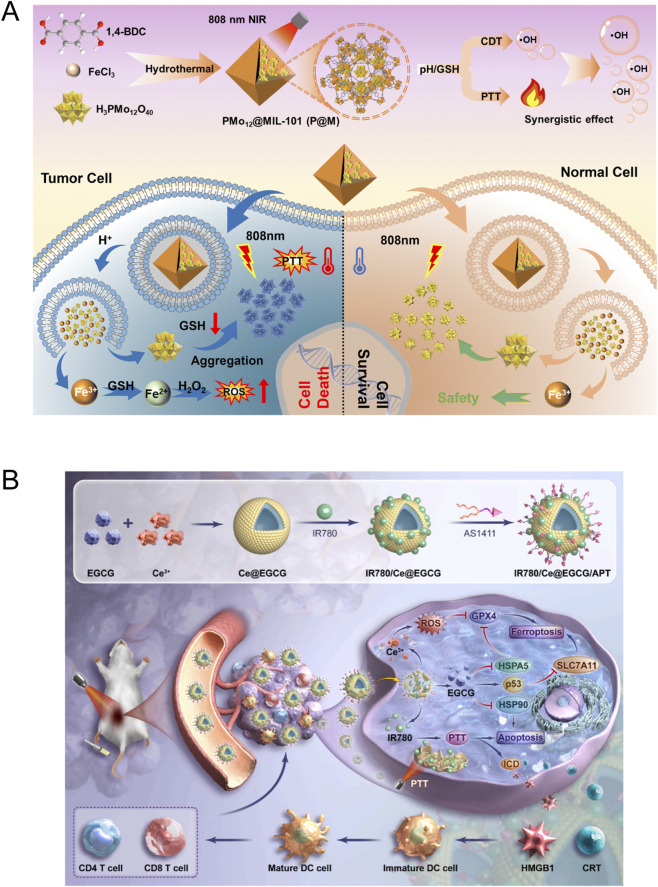
**(A)** Schematic illustration of tumor cell-specific GSH/pH dual activated P@M for combined PTT/chemodynamic therapy treatment. **(B)** Schematic illustration of IR780/Ce@EGCG/APT preparation and the mechanism of synergistic therapy (Li et al., 2024a; Mu et al., 2024).

Metal-based nanomaterials, owing to their tunable dimensions, shapes, and surface functionalization, serve as ideal carriers for PTAs in PTT and are rapidly advancing in the field of PTAs’ carriers. For example, Li et al. (Li et al., 2024a) designed a TME-responsive PMo_12_@MIL-101 (POM@MOF) by loading polyoxometalates (POMs) onto Fe- and Cu-based MOFs. Molybdenum-containing POMs were nanoscale inorganic polymetallic oxygen clusters that exhibited strong NIR absorption when reduced to molybdenum blue. The highly negatively charged surface of POMs hindered their penetration through cell membranes. MOFs loading modified their surface charge to enhance uptake by tumor cells. Subsequently, under the tumor’s acidic environment and GSH influence, they reduced to PMo_12_ and Fe^2+^, exhibiting excellent PCE (47.03%) upon NIR irradiation. The mechanism of action for POM@MOF is illustrated in Figure 5A. Xie et al. (2024) prepared nano-TiO_2_-coated MCNTs by encapsulating TiO_2_ nanomaterials within multi-walled carbon NTs (MCNTs). MCNTs inherently exhibited significant light absorption within the 808 nm–1064 nm NIR window, and this absorption effect was further intensified upon modification with TiO_2_ nanomaterials. This manifested as a significant reduction in the expression of cyclin CCNA1 and CCND1 in colorectal cancer cells under NIR irradiation, indicating that TiO_2_ NS-modified MCNTs could potentially serve as an effective strategy for PTT treatment of colorectal cancer.

Secondly, Mu et al. (2024) utilized rare earth element cerium (Ce) to encapsulate IR780 within MPN, synthesizing IR780/Ce@EGCG. The synthesis process and mechanism of action for IR780/Ce@EGCG are illustrated in Figure 5B. Here, epigallocatechin gallate (EGCG) reduced heat shock protein production to enhance PTT efficacy; Ce generated ROS via a Fenton-like reaction, jointly inducing ferroptosis in tumor cells; while IR780 accumulated as PTA in tumor cell mitochondria, generating photothermal effects under 808 nm NIR irradiation to induce tumor cell death. Cun et al. (2022) designed an IR780-loaded Fe_3_O_4_@MIL-100 NPs (IFM) based on Fe. MIL-100 was a MOF. Fe_3_O_4_ NPs modified with MIL-100 exhibited enhanced peroxidase activity and GSH consumption capacity, enabling IFM to generate more highly cytotoxic ·OH radicals and kill tumor cells via ferroptosis. IFM demonstrated highly efficient tumor cell killing via combined ferroptosis and PTT therapy, achieving a tumor inhibition rate of 96.4%.

In summary, metal-based nanomaterials demonstrate tunable optical absorption, photothermal synergistic effects, excellent biocompatibility, and multimodal diagnostic and therapeutic capabilities. They overcome many limitations of traditional PTAs, such as poor water solubility and low photostability, making them highly potential candidate materials in the future PTT anti-tumor field.

#### 3.2.2 Metal-based nanomaterials as PTAs

When metal-based nanomaterials are applied as PTAs, precious metals such as Au and Ag exhibit superior LSPR and PCE compared to other metallic materials. Consequently, these precious metal nanostructures have become the mainstream in this field (Liu et al., 2016; Sarfraz et al., 2018; Gellé et al., 2020; Wang et al., 2022a; Li et al., 2025b). The following sections will discuss the applications of Au and Ag nanostructures as well as other metal-based nanomaterials in PTAs.

##### 3.2.2.1 Au nanomaterials

**FIGURE 6:**
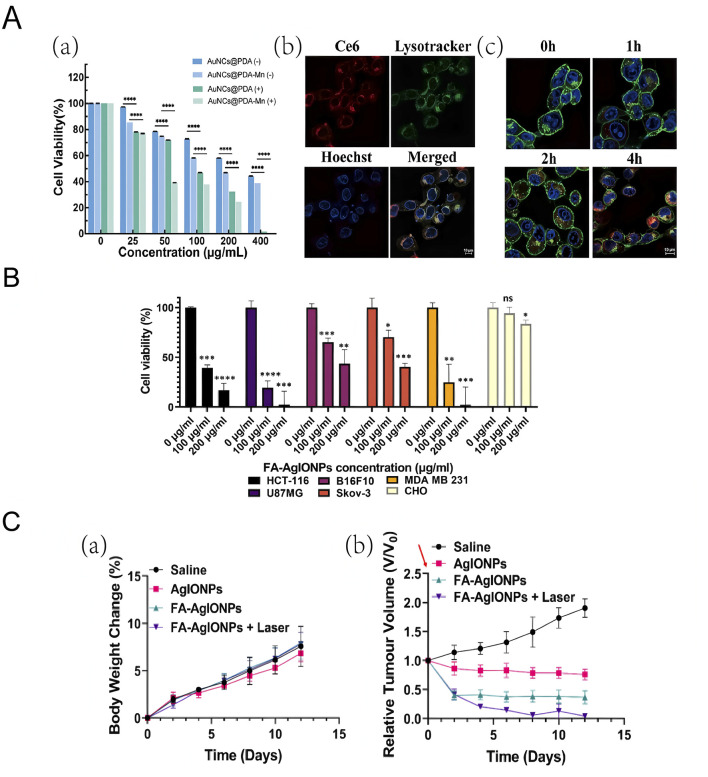
**(A)** (a) Relative cell viability of 4T1 cells after incubation with different concentrations of AuNCs@PDA and AuNCs@PDA-Mn in the absence or presence of light irradiation (1.0 W cm^-2^, 5 min). (b) CLSM images of 4T1 cells incubated with AuNCs@PDA-Mn for 4 h. Blue fluorescence shows nuclear from Hoechst; red fluorescence is attributed to Ce6 labelled AuNCs@PDA-Mn; green fluorescence shows lysosomes from Lysotracker Green (Lyso Green). (c) CLSM images of the cellular uptake of AuNCs@PDA-Mn at different times. **(B)** A panel of cancer cell lines was treated with AgIONPs with the FA ligand at varying NP concentrations. **(C)** (a) Average body weight of mice was monitored twice a week during the treatment. (b) Changes in tumor volume during the experiment period. Relative tumor volume was defined as the tumor volume (V) measured normalized to the tumor volume on day 0 before administration of treatment (V0). The red arrow denotes initiation of injection of NPs (Moonshi et al., 2023; Yang et al., 2025).

In the field of PTAs, Au nanomaterials can serve not only as carriers for PTAs but also directly as PTAs themselves due to their excellent LSPR properties. For instance, Yang et al. (2025) designed an MPN-coated AuNCs@PDA-Mn for Au NCs, demonstrating outstanding breast cancer cell killing ability (Figure 6A). Au NCs, composed of several to hundreds of Au atoms, exhibit advantages such as a relatively stable multinuclear aggregate structure and good water solubility. After 10 min of 808 nm laser irradiation, AuNCs@PDA-Mn effectively suppressed tumor growth. The immunogenic cell death (ICD) induced by PTT further enhanced the body’s antitumor immune function. Since the toxicity of Au nanomaterials correlates with particle size, Song et al. (2015) aimed to reduce toxicity while fully leveraging Au’s superior properties. They coated the surfaces of 8 × 2 nm Au NRs with hydrophilic PEG and hydrophobic poly (lactic-co-glycolic acid) (PLGA), yielding AuNR@PEG/PLGA particles with ultra-small dimensions (approximately 60 nm). This formulation self-assembled into plasmonic vesicles. Due to their ultra-small size, the Au NRs were rapidly cleared *in vivo*; most vesicles were eliminated from the mouse body within 10 days post-injection. AuNR@PEG/PLGA exhibited advantages such as extended circulation time *in vivo*, significant accumulation in tumor tissues, and enhanced photothermal performance. Free Au NRs also demonstrated excellent PCE and thermal ablation effects under low-intensity NIR irradiation. Furthermore, Zhao et al. (Zhao et al., 2021) modified Au NRs with a high aspect ratio using BSA to create AuNR@BSA, significantly reducing the Au NRs’ cytotoxicity. This modification enabled PTT activation even under weak light irradiation, effectively inhibiting tumor growth.

##### 3.2.2.2 Ag nanomaterials

Ag nanomaterials exhibit outstanding optical properties and narrow emission peaks, with their LSPR effect significantly surpassing that of other metallic nanomaterials (Jaque et al., 2014). Consequently, they demonstrate exceptional photothermal performance and possess potent antibacterial and anticancer effects. The PTT effect of Ag nanomaterials were first demonstrated by Shipunova et al. (Shipunova et al., 2022). They designed a HER2-targeted Ag NPs, Ag-PEG-HER2, with a diameter of approximately 35 nm. This NPs utilized Z_HER2:342_ for targeted delivery to tumor tissues, subsequently killing tumor cells via PTT under 465 nm light irradiation. Aiello et al. (Rivas Aiello et al., 2021) synthesized vinylpyrrolidone-coated Ag NPs, PVPAgNPs, exhibiting absorption peaks between 650 nm and 1450 nm. Under NIR irradiation, these NPs demonstrated excellent photothermal effects, effectively generated plasmons and rapidly killed tumor cells. Moonshi et al. (Moonshi et al., 2023) designed AgIONPs based on Ag and Fe_2_O_3_, which were modified with folic acid (FA) to yield FA-AgIONPs. These exhibited outstanding antitumor efficacy both *in vivo* and *in vitro* (Figures 6B,C), attributed to the combined anticancer effects of Ag itself and the photothermal effect induced by NIR irradiation. Beyond PTT applications, FA-AgIONPs also possessed MRI imaging capabilities, demonstrating broad prospects in both tumor PTT and imaging diagnostics.

##### 3.2.2.3 Other precious metal-based nanomaterials

Beyond Au and Ag nanomaterials, other precious metal-based nanomaterials have been employed for PTT. For instance, Li et al. (2022a) developed a nanocomposite based on ruthenium (Ru) and Au, DMSN-Au-Ru NPs. This material not only possessed the properties of Au nanomaterials but also catalyzed glucose conversion to hydrogen peroxide via glucose oxidase (GOD). This process generated ^1^O_2_ under Ru nanomaterials decomposition, thereby enhancing PTT efficacy. Zhao et al. (Zhang et al., 2023c) prepared Bre-YAP using Pt and Au NPs via ultrasonic disruption and differential centrifugation. This formulation exhibited high-level distribution in both tumor tissues and tumor-draining lymph nodes, enhancing the efficacy of chemotherapeutic and PTT.

Ma et al. (2022) designed NMOF545@Pt, which leveraged the presence of Mn to shorten longitudinal relaxation time (T1), enhanced the photoacoustic effect, and effectively increased X-ray absorption. Under 808 nm light irradiation, NMOF545@Pt significantly shrinked tumors. Due to its high X-ray absorptivity, nearly no tumor cells survived under combined PTT and radiotherapy, with no significant organ damage observed in healthy tissues. This demonstrated that NMOF545@Pt not only exhibited potent antitumor effects but also possessed excellent biocompatibility, making it a promising candidate for highly effective nanotherapeutic agents. Additionally, Liu et al. (2024a) designed a novel nanomaterial, Fn@Pt (FP), using Pt, Mn, and ferritin (Fn) as raw materials, which exhibited excellent biocompatibility and photothermal effects.

##### 3.2.2.4 Other metal-based nanomaterials

Non-precious metal-based nanomaterials, while lacking the superior LSPR and PCE characteristics of precious metals, remain indispensable components in the PTAs field due to their advantages such as low cost and high production volume. Examples include Cu, Fe, Mn, Ti, nickel (Ni), cobalt (Co), tungsten (W), bismuth (Bi), hafnium (Hf), etc. (Li et al., 2023). For example, Ma et al., 2020) designed a bismuth-based nanocarrier that, when combined with chemotherapy drugs, formed the nanomedicine Pt@Bi_2_Te_3_-PEG NPs. This formulation exhibited strong NIR absorption properties, achieved a PCE of 48.7%, and could also serve as a contrast agent for bioimaging. Pt@Bi_2_Te_3_-PEG NPs enhanced tumor cell uptake of Pt, enabling combined PTT and chemotherapy for cancer treatment. This approach showed promising applications in combined cancer therapy and bioimaging. Xiong et al. (2021) similarly prepared Bi_19_S_27_I_3_ NRs as PTAs via a simple solvothermal method based on bismuth. Bi_19_S_27_I_3_ NRs exhibited exceptionally high PCEs of 42.7% and 41.5% in NIR-I and NIR-II regions, respectively. The resulting thermal ablation completely eradicated tumor tissue, demonstrating the excellent application prospects of PTT in tumor therapy.

Geng et al. (2022) synthesized a Cu-MOF embedded with CuS (CuS@Cu-MOF) exhibiting excellent photothermal effects. By elevating the temperature, the rate of the Cu-driven pseudo-Fenton effect could be significantly enhanced. Additionally, DOX could be encapsulated within the MOF, enabling further tumor tissue destruction through the combination of chemotherapy and PTT. TiN is a promising alternative to Au, exhibiting excellent photostability and biocompatibility while strongly absorbing NIR light (Guler et al., 2015; He et al., 2017). Popov et al. (2019) investigated TiN-based nanomaterials, co-cultivated cancer cells with TiN NPs and exposed them to NIR irradiation. After four irradiation sessions, the high temperatures generated by TiN NPs caused significant tumor cell death. Pastukhov et al. (2024) introduced Hf-based HfN NPs, achieving a PCE of 62% under 808 nm light irradiation. Results showed that PEG-modified PEG-HfN NPs at a concentration of 25 μg mL^-1^ completely ablated tumor cells upon 808 nm light irradiation. The applications of precious metal-based and non-precious metal-based nanomaterials in PTT are detailed in Table 2.

**TABLE 2:** Application of metal-based nanomaterials in PTT.

Metal type	Name	Photothermal efficacy	Light source	*In vitro* anti-cancer effect	*In vivo* anti-cancer effect	Biosafety	References
Au	AuNCs@PDA-Mn	In the wavelength of 808 nm, with a power density of 1.0 W cm^-2^, the temperature of 200 μg mL^-1^ AuNCs@PDA-Mn could rise by 17.5 °C when subjected to continuous radiation	*In vitro* and *in vivo*: 808 nm	As the concentration of AuNCs@PDA-Mn increased from 0 μg mL^-1^–400 μg mL^-1^, nearly all 4T1 cells were killed	After 14 days of cultivation, AuNCs@PDA-Mn exhibited a significant inhibitory effect on tumor growth and metastasis in 4T1-bearing mice	The results of the blood biochemical analysis demonstrated that there were no significant changes in the levels of organ biomarkers on day 14 following the administration of the final injection	Yang et al. (2025)
​	AuNR@PEG/PLGA	The temperature of the vesicle solution (0.1 × 10–^9^ _M_ AuNR@PEG/PLGA) rapidly reached 75.2 °C after irradiation with 808 nm laser (0.8 W cm^-2^) for 5 min	*In vitro* and *in vivo*: 808 nm	After adding AuNR@PEG/PLGA and treating with an 808 nm laser for 5 min, over 90% of U87MG human glioma cells were killed	After 15 days of cultivation, all tumor cells in the U87MG tumor mice were eliminated, with survival times ranging from 40 to 50 days	No obvious infl ammation or damage of major organs of mice, including heart, liver, spleen, lung, and kidneys, treated with vesicles and laser on day 10	Song et al. (2015)
​	AuNR@BSA	Under 1064 nm laser irradiation, the temperature of AuNR@BSA-treated 4T1 cells rapidly increased during the first 5 min and then remained at approximately 80 °C for the remainder of the exposure period	*In vitro* and *in vivo*: 1064 nm	As the absorbance of the AuNR@BSA solution increased from 0 to 2, the 4T1 cell survival rate decreased to 20%	After 14 days of treatment, tumors in 4T1-bearing mice treated with AuNR@BSA and 1064 nm laser irradiation were completely suppressed, with eschar formation occurring at the tumor site	The biocompatibility of the reported AuNR was significantly improved by coating with BSA, and the photothermal properties were not affected	Zhao et al. (2021)
Ag	Ag-PEG-HER 2	The highest temperature value was achieved at an LED matrix power of 95 mW cm^-2^ after 25 min of irradiation at a NP concentration of 2.2 mg mL^-1^ and exceeded the buffer temperature change by 10 °C	*In vitro* and *in vivo*: 465 nm	As the concentration of Ag-PEG-HER2 increased from 0 mg mL^-1^–10 mg mL^-1^, nearly all HER2-overexpressing SKOV3-1ip and HER2-negative CHO cells were killed	Following over 90 days of cultivation, *in vivo* bioimaging studies in tumor-bearing mice demonstrated that light therapy administered after Ag-PEG-HER2 treatment not only resulted in complete elimination of the primary tumor but also prevented metastatic spread	Histological research results indicate that mild lesions were observed in the lungs and kidneys of mice in the experimental group, indicating certain deficiencies in biological safety	Shipunova et al. (2022)
​	FA-AgIONPs	After 5 min of exposure to 808 nm laser irradiation, the temperature of the 50 mg mL^-1^ FA-AgIONPs solution rose to 72 °C	*In vitro* and *in vivo*: 808 nm	The temperature increment with FA-targeted NPs was 12 °C higher than the nontargeted group, suggesting better uptake of targeted NPs, which led to a higher temperature elevation. Consequently, exposure to laser irradiation resulted in cell viabilities of 0% and 32.9% in targeted and nontargeted groups	After 13 days of cultivation, it was observed that a single intravenous dose of 10 mg kg^-1^ FA-AgIONPs combined with a single 5 min laser irradiation session at 1.5 W cm^-2^ produced effective photothermal tumor ablation in HeLa tumor-bearing mice	Histological images of tissues of major organs stained with H&E revealed no noticeable tissue damage, inflammation, or lesion at the highest NP concentration (10 mg kg^-1^ of Ag) in comparison to the control group	Moonshi et al. (2023)
​	PVPAgNP	Under 800 nm laser irradiation, the temperature of the PVPAgNP suspension monotonically increased and stabilized after approximately 30 min	*In vitro*: 640 nm	PVPAgNPs absorbed by cells enabled their destruction through red light irradiation, while remaining harmless to cells without PVPAgNPs	N/A	N/A	Rivas Aiello et al. (2021)
Fe	POM@MOF	Under conditions of pH 5.5, when irradiated with 808 nm laser light, the temperature of a 1000 μg mL^-1^ POM@MOF solution rose to 70 °C within 2 min	*In vitro* and *in vivo*: 808 nm	As the concentration of POM@MOF increased from 0 μg mL^-1^ –200 μg mL^-1^, the survival rate of HepG2 cells in the H_2_O_2_-treated group decreased to 14.8%	N/A	N/A	Li et al. (2024a)
​	Fe_3_O_4_@MIL-100	After 10 min of irradiation with an 808 nm laser, the temperature of the 100 μg mL^-1^ Fe_3_O_4_@MIL-100 solution increased by 42.8 °C	*In vitro* and *in vivo*: 808 nm	As the Fe_3_O_4_@MIL-100 concentration increased from 3.2 μg mL^-1^–50 μg mL^-1^, the activity of 4T1 cells decreased to approximately 5%	After 14 days of treatment, Fe_3_O_4_@MIL-100 (4 mg mL^-1^, 100 μL) achieved a tumor inhibition rate of 96.4% in 4T1 tumor-bearing mice, with 2 out of 5 mice exhibiting complete tumor regression	The main blood parameters showed no significant changes compared to those of control group, indicating that blood compatibility of Fe_3_O_4_@MIL-100. H&E staining of key organs assay indicated no evident damage or inflammatory lesions during therapy	Cun et al. (2022)
Ti	nano-TiO_2_-coated MCNTs	After 10 min of exposure to 808 nm laser irradiation, the temperature of the 200 μg mL^-1^ MCNTs solution rose to 59 °C	*In vitro* and *in vivo*: 808 nm	As the concentration of MCNTs increased from 0 μg mL^-1^–200 μg mL^-1^, the viability of HCT116 and HT-29 cells decreased to approximately 30%	After 20 days of cultivation, the relative tumor volume and tumor mass in HCT116-bearing mice treated with 100 μg/mL MCNTs were the lowest	N/A	Xie et al. (2024)
Ce	IR780/Ce@EGCG/APT	A solution with a concentration of 1 μg mL^-1^ of IR780/Ce@EGCG/APT exhibited a PCE of 70.76% under laser irradiation at 808 nm	*In vitro* and *in vivo*: 808 nm	As the concentration of IR780/Ce@EGCG/APT increased from 0.25 μg mL^-1^–3 μg mL^-1^, the activity of 4T1 cells decreased to approximately 10%	After 21 days of cultivation, treatment with 1.0 mg mL^-1^ IR780/Ce@EGCG/APT significantly inhibited tumor growth in 4T1-bearing mice. Furthermore, when 4T1 cells were re-injected on day 28, the mice exhibited slower tumor growth, suggesting that the treatment stimulated the mice’s defense against 4T1 cells	The body weights of mice after treatment with different formulations showed an upward trend. The H&E staining of the major organs displayed no obvious pathological alterations compared with the NS group, indicating the negligible systemic toxicity of IR780/Ce@EGCG/APT(+L) nanoreactor	Mu et al. (2024)
Ru	DMSN-Au-Ru NPs	The temperature of DMSN-Au-Ru NPs rose rapidly with the increasing of the concentration and time extension, and the PCE under 808 nm irradiation was calculated to be 36.43%	*In vitro* and *in vivo*: 808 nm	As the concentration of DMSN-Au-Ru NPs increased from 0 μg mL^-1^ –200 μg mL^-1^, over 90% of HeLa cells were killed	In U14 tumor-bearing mice treated with DMSN-Au-Ru NPs and 808 nm laser irradiation, complete tumor ablation was achieved by day 5, with no recurrence observed during the subsequent 9-day follow-up period	The body weight of the mice in all groups did not change significantly during the 14 days. No significant damage was observed in the main organs of the mice in the experimental group through H&E staining, and there were no notable changes in blood indicators after 30 days compared to the control group mice	Li et al. (2022a)
Pt	Bre-YAP	After 5 min of exposure to 808 nm laser irradiation, the temperature of the 0.4 mg mL^-1^ Bre-YAP solution rose and remained at 62.6 °C	*In vitro* and *in vivo*: 808 nm	As the concentration of Bre-YAP increased from 0 μg mL^-1^ –2 μg mL^-1^, the activity of B16F10 cells gradually decreased to approximately 30%	Following treatment with 50 μg of Bre-YAP and 2 min of 808 nm laser irradiation, B16F10 tumor-bearing mice exhibited the longest survival time of approximately 28 days, with the most pronounced nuclear fragmentation observed in hematoxylin and eosin (H&E)-stained tumor sections	No significant damage was observed in the main organs of the mice in the experimental group through H&E staining	Zhang et al. (2023c)
​	NMOF545@Pt	Under 808 nm laser irradiation, the temperature of a 200 μg mL^-1^ NMOF545@Pt solution increased from 25.4 °C to 57.6 °C	*In vitro* and *in vivo*: 808 nm	As the concentration of NMOF545@Pt increased from 0 μg mL^-1^ –400 μg mL^-1^, the viability of 4T1 cells decreased to approximately 20% following irradiation with an 808 nm laser and 4 Gy of X-rays	After 14 days of cultivation, tumor growth in NMOF545@Pt-treated tumor-bearing mice was significantly suppressed under 808 nm laser irradiation and 4 Gy X-ray exposure, with virtually no viable tumor cells remaining	Compared with the control group, no significant changes were observed in the body weight and H&E staining of major organs in the experimental group mice	Ma et al. (2022)
​	Fn@Pt (FP)	At a concentration of 160 μg mL^-1^ in the Fn@Pt (FP) solution, the PCE of Fn@Pt (FP) under 808 nm laser irradiation was 26.25%	*In vitro* and *in vivo*: 808 nm	As the concentration of Fn@Pt (FP) increased from 0 μg mL^-1^ –40 μg mL^-1^, the activity of 3T3 cells gradually decreased to approximately 40%	After 14 days of cultivation, the relative tumor volume and weight were minimal in MDA-MB-231 tumor-bearing mice treated with 808 nm laser and Fn@Pt (FP)	The hemolysis tests verified the biocompatibility of Fn@Pt (FP) *in vitro*. The average hemolysis rate of Fn@Pt (FP) group was less than 5%, so Fn@Pt (FP) possesses good biocompatibility. H&E staining suggested that there is no observable toxicity resulting from Fn@Pt (FP)	Liu et al. (2024a)
Bi	Pt@Bi_2_Te_3_-PEG NPs	After irradiation with an 808 nm laser, the PCE of an 80 μg mL^-1^ Pt@Bi_2_Te_3_-PEG NPs solution reached 48.7%	*In vitro* and *in vivo*: 808 nm	As the concentration of Pt@Bi_2_Te_3_-PEG NPs increased from 0 μg mL^-1^ –50 μg mL^-1^, the mortality rate of 4T1 cells reached 74.2% ± 2.8%	After 14 days of cultivation, treatment with Pt@Bi_2_Te_3_-PEG NPs (100 μL, 2 mg mL^-1^) resulted in the smallest relative tumor volume in 4T1 tumor-bearing mice, demonstrating near-complete tumor eradication	Compared with the control group, no significant changes were observed in the body weight, hematological parameters, and H&E staining of major organs in the experimental group mice	Ma et al. (2020)
​	Bi_19_S_27_I_3_ NRs	At a concentration of 500 μg mL^-1^, the PCEs of Bi_19_S_27_I_3_ NRs at 808 nm and 1064 nm were 42.7% and 41.5%, respectively	*In vitro* and *in vivo*: 808 nm or 1064 nm	As the Bi_19_S_27_I_3_ NRs concentration increased from 0 μg mL^-1^ –500 μg mL^-1^, HeLa cell viability decreased to approximately 30% after 5 min of laser irradiation at either 808 nm or 1064 nm. Cell killing efficiency was significantly higher under NIR-1064 irradiation compared to NIR-808 irradiation	Following the injection of 0.2 mL Bi_19_S_27_I_3_ NRs into HeLa tumor-bearing mice, exposure to NIR-808 and NIR-1064 irradiation rapidly elevated body temperatures to 45.1 °C and 53.7 °C, respectively, within 10 min. After 14 days of follow-up, these mice exhibited the lowest relative tumor volumes	Compared with the control group, no significant changes were observed in the body weight in the experimental group mice	Xiong et al. (2021)
Hf	HfN NPs	At a concentration of 100 μg mL^-1^, the PCE of HfN NPs could reach 62% under irradiation with an 808 nm laser	*In vitro*: 808 nm	Even at a concentration of 10 μg mL^-1^ for HfN NPs, the metabolic activity of BT474 and EMT6/P cells was reduced by 15% and 24%, respectively. With further increased in concentration, the cells were nearly completely killed	N/A	N/A	Pastukhov et al. (2024)

In summary, metal-based nanomaterials can serve as carriers for PSs/PTAs or function directly as PSs/PTAs in PT, and exhibit significant advantages over traditional materials (Table 3). When acting as carriers, they significantly enhance the water solubility, photostability, and tumor accumulation of PSs/PTAs. When acting directly as PSs/PTAs, they improve PT efficacy through intrinsic properties such as the LSPR and superior PCE. The rapid advancement of metal-based nanomaterials provides a robust material foundation for achieving efficient, controllable PT with strong potential for clinical translation.

**TABLE 3:** Performance comparison between traditional materials and metal-based nanomaterials.

Benchmark	Traditional materials	Metal-based nanomaterials	References
Photostability	Poor, prone to photobleaching	Excellent, resistant to long-term exposure	Shamsian et al. (2025)
PCE	15%–25%	30%–80% higher than traditional materials	Yu et al. (2024)
^1^O_2_ yield	30%–40%	80%–150%	Younis et al. (2021)
Tumor accumulation efficiency	Low, non-specific distribution	High, enhanced permeability and retention effect + tumor targeting	Han et al. (2025)
Surface modifiability	Moderate	Excellent	Yang et al. (2025)

## 4 Design and optimization of metal-based nanomaterials

### 4.1 Structural design of metal-based nanomaterials

As mentioned above, different metals exhibit distinct effects, thereby influencing PT efficacy in varying ways. Additionally, optimizing PT efficacy can be achieved by adjusting the physicochemical parameters of metal-based nanomaterials. The three most critical parameters are size, shape, and surface functionalization (Dawi et al., 2020; He et al., 2021; Zhang et al., 2023a; Liu et al., 2025). We will now discuss the influence of each of these key parameters on PT efficacy.

#### 4.1.1 Effect of size on the efficacy of PT in metal-based nanomaterials

Size is the most fundamental parameter of metal-based nanomaterials. By controlling size, the characteristics of LSPR can be preliminarily adjusted to enhance the therapeutic efficacy of PT. Its effects are primarily reflected in the peak position and width of LSPR absorption, the absorption-to-scattering ratio, and biological toxicity (Dreaden and El-Sayed, 2012; Lin et al., 2013). For metal-based nanomaterials smaller than 20 nm, the LSPR peak position shows low sensitivity to size variations and is instead influenced by factors such as shape and the metal matrix. In contrast, for medium to large metal-based nanomaterials exceeding 20 nm, increasing size results in a significant red shift of the LSPR peak position (Kolosnjaj-Tabi et al., 2019; Wen et al., 2020). For example, for Au NPs larger than 20 nm, the wavelength of their LSPR absorption peak exhibits a clear positive correlation with particle diameter. As the size of Au NPs increases from 22 nm to 99 nm, the maximum absorption wavelength shifts from approximately 500 nm to nearly 600 nm. This shift may occur because, as the size of Au NPs increases, the propagation of electromagnetic waves within the particle induces a phase delay effect. Concurrently, size-dependent multipole resonances become more pronounced, collectively causing a red shift in the LSPR absorption peak (Li et al., 2016). The therapeutic window for NIR spans 700 nm–900 nm. To achieve this window solely through size variation, Au NPs must exceed 100 nm in diameter. However, this results in drawbacks such as increased scattering effects, reduced biocompatibility, and compromised stability (Park et al., 2016; Zhang et al., 2019a; Bai et al., 2020). Therefore, merely adjusting the size of metal-based nanomaterials is insufficient for achieving more efficient and safer PT.

#### 4.1.2 Effect of shape on the efficacy of PT in metal-based nanomaterials

The shape of metal-based nanomaterials is also a key factor influencing PT efficacy (Yang et al., 2022). Metal-based nanomaterials exhibit excellent ductility and tunability, allowing them to be engineered into diverse shapes. For example, Au nanomaterials can be designed as nanostars, NRs, NSPs, NCs, nanoshells, and nanoblossoms, exhibiting specific PCE ranging from 22% to 103% (Feng et al., 2024). Among these, Au NSPs exhibit only one relatively narrow LSPR peak centered around 520 nm, but their LSPR peaks undergo red/blue shifts upon modification (Wang et al., 2012; Zhang et al., 2019b; Akasov et al., 2022). For example, Ye et al. (Ye et al., 2014) modified Ag NPs with Au NSPs to obtain Au@Ag NSPs. This formulation exhibited a broad and intense LSPR peak in the 400 nm–1100 nm range and effectively killed lung cancer cells upon irradiation at 980 nm. When metal-based nanomaterials are engineered as NRs, dual LSPR peaks emerge: a longitudinal LSPR peak and a transverse LSPR peak. Taking Au NRs as an example, their transverse LSPR peak is relatively fixed at approximately 520 nm. The longitudinal LSPR peak corresponds to electron oscillations along the long axis and is highly sensitive to changes in aspect ratio (AR). An increase in AR causes a significant red shift in the longitudinal LSPR peak. Therefore, by controlling AR, the longitudinal LSPR can be shifted to the NIR-I or even NIR-II windows (Sun et al., 2017), effectively addressing the challenge of NSP absorption peaks entering the NIR window. With their numerous advantages, Au NRs have become one of the most extensively studied and successfully applied metal-based nanomaterials.

Additionally, other modulation strategies exist for more complex metal-based nanomaterials. Taking Au nanocages as an example, their absorption peak position is primarily determined by the ratio of cage wall thickness (t) to overall edge length (L) (t/L). The general trend shows that as the t/L ratio decreases, the absorption peak of Au nanocages undergoes a significant red shift (Zhang et al., 2021; Li et al., 2024b). For instance, when the cage wall thickness t decreases from 20 nm to 5 nm, the absorption peak shifts from approximately 800 nm–1100 nm (Li and Gu, 2009). Similarly, when the edge length L increases from 45–50 nm to 80–100 nm, the absorption peak shifts to 900–1000 nm (Xu et al., 2019).

Although shaping metal-based nanomaterials can enhance PTT efficacy, relying solely on morphology control presents significant limitations. For example, complex shapes often require more intricate fabrication processes, and performance variations frequently occur between different batches (Xie et al., 2021b; Tang et al., 2022; Zeng et al., 2024b). Therefore, more precise fabrication techniques are required to maintain the consistency of nanomaterials, ensuring the stability and reproducibility of their photothermal therapeutic effects. Some researchers have proposed using capping agents to preserve morphological stability during the large-scale synthesis of nanomaterials. For example, cetyltrimethylammonium bromide (CTAB), a halide compound, has been shown to play a crucial role in regulating the morphology of various nanomaterials (Liu et al., 2013; Wu and Yang, 2017). Additionally, different shapes of nanomaterials can be obtained by adjusting the concentration ratios of the raw materials. For instance, during the synthesis of Au-based nanomaterials, varying the concentration ratios of Au salt, Ag nitrate, and ascorbic acid enables the preparation of Au NSPs, pentagonal twin Au NRs, and trioctahedral Au NPs, respectively (Lohse et al., 2014; Zhang et al., 2019c).

Secondly, the stability of nanomaterials cannot be overlooked. Although morphologies with high AR and low t/L ratio can red-shift LSPR peaks into the NIR therapeutic window, they are prone to deformation in physiological environments, which compromises therapeutic efficacy. Additionally, nanomaterials with sharp surfaces, such as Au NCs and Au nanostars, can cause inflammation by damaging cell membranes, resulting in poor biocompatibility and high toxicity (Yoo et al., 2016). Therefore, to fully leverage the PT antitumor effects of metal-based nanomaterials while addressing the challenges associated with morphology adjustments, surface functionalization remains essential.

#### 4.1.3 Effect of surface functionalization of metal-based nanomaterials on PT efficacy

Surface functionalization of metal-based nanomaterials involves the application of specific molecular or polymer coatings onto their surfaces. This strategy addresses challenges such as rapid clearance by the reticuloendothelial system, poor biocompatibility, and limited accumulation within tumor tissues (Yuan et al., 2012). For instance, after intravenous injection, metal-based nanomaterials rapidly adsorb plasma proteins to form a “protein corona” (PC), making them more susceptible to recognition and clearance by macrophages. This shortens their half-life (Xiao et al., 2022), ultimately compromising the PT effect. PEGylation of the nanomaterials effectively resolves this issue. For instance, Liu et al. (Liu et al., 2014) improved PC formation by introducing high-density PEG chains onto superparamagnetic Fe_2_O_3_ NPs (SPIONs). Animal studies demonstrated that PEGylated SPIONs exhibited a half-life of 204 min, significantly longer than that of SPION-PAA (37 min), laying a solid foundation for ensuring PT efficacy. Additionally, Lankveld et al. (Lankveld et al., 2011) extended the half-life of Au NRs from 15 min to 17–19 h through PEGylation.

**FIGURE 7:**
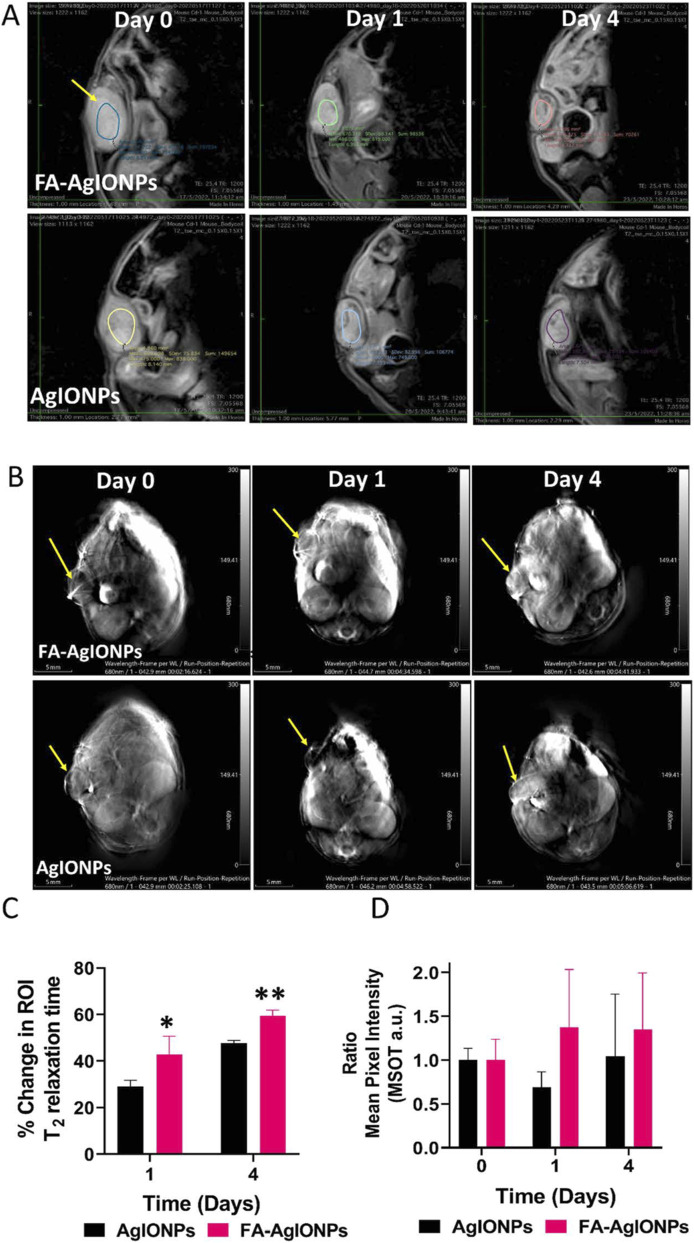
*In vivo* tumor targeting (MRI T2 contrast and PAI) of AgIONPs and FA-AgIONPs in U-87 MG tumor-bearing BALB/C mice. Mice were injected intravenously with 10 mg kg^-1^ (Ag) NPs with and without FA targeting. Mouse tumors were imaged before (day 0), 1 day, and 4 days post injection of NPs. **(A)** Representative T2*-weighted images of mouse tumors using a 7 T MRI. **(B)** Representative MSOT images. **(C)** Quantitative analysis of % change in the ROI T2 relaxation time of tumors injected with AgIONPs and FA-AgIONPs. **(D)** Quantitative ratiometric analysis of the MSOT mean pixel intensity at 680 nm from the center of the tumors injected with AgIONPs and FA-AgIONPs (Moonshi et al., 2023).

Surface functionalization not only prolongs the *in vivo* half-life of metal-based nanomaterials but also significantly enhances their tumor-targeting capabilities. Tumor tissues commonly overexpress folate receptors (FRs), particularly the FRα subtype (Matulonis et al., 2023). Leveraging this biological characteristic, researchers have achieved precise targeting of FRα-positive tumor cells by conjugating FR ligands to the surface of nanomaterials. For example, as mentioned earlier, Moonshi et al. (Moonshi et al., 2023) first introduced sulfur-containing PEG chains onto the NPs surface, then covalently linked FA to the PEG-SH chains via amide bonds. This configuration allowed FA to adopt a rotatable segment, ultimately yielding FA-AgIONPs. Flow cytometry and TEM results demonstrated that FA-modified NPs exhibited significantly higher uptake in glioblastoma cells at both the 4 h and 24 h time points compared to unmodified AgIONPs, while FA-AgIONPs did not accumulate in normal tissues (Figure 7). This targeted strategy not only enhanced the local concentration of NPs at tumor sites but also reduced side effects on normal tissues. Additionally, Xiao et al. (2011) noted that positively charged metal-based nanomaterials more readily bound to negatively charged cell membranes via electrostatic interactions and were more easily cleared by the reticuloendothelial system, resulting in shorter circulation times. In contrast, neutral or negatively charged metal-based nanomaterials were more difficult to clear. Therefore, extending the *in vivo* circulation time can be achieved by surface-modifying metal-based nanomaterials with negatively charged functional groups.

In summary, surface functionalization effectively addresses the challenge of ineffective *in vivo* treatment by regulating the half-life of metal-based nanomaterials, enhancing tumor-targeting capabilities, and optimizing surface charge distribution. However, research on the underlying mechanisms through which surface functionalization improves the photothermal performance of metal-based nanomaterials remains limited, particularly concerning surface charge regulation, where the relevant mechanisms are still unclear. Although surface functionalization shows promise for safer and more efficient enhancement of PT efficacy, this area requires more systematic and in-depth exploration.

From precise dimensional control and morphology optimization to surface functionalization, a range of strategies is driving the advancement of metal-based nanomaterials toward greater precision, efficiency, and safety. With continuous improvements in synthetic preparation techniques and deeper insights into the intrinsic mechanisms governing interactions between nanomaterials and biological systems, it will become feasible to design and construct next-generation metal-based nanomaterials that combine high therapeutic efficacy, strong tumor specificity, and excellent biosafety. This progress will provide robust technological support for enhancing PT efficacy and addressing the challenges posed by malignant tumors.

### 4.2 Responsive design of metal-based nanomaterials

The microenvironment of tumor tissues differs significantly from that of normal tissues, characterized by hypoxic conditions, elevated H_2_O_2_ concentrations, a mildly acidic environment (pH = 6.4–6.8), high GSH levels, and locally elevated temperatures. These features facilitate rapid tumor proliferation and immune evasion while providing natural responsive targets for the design of metal-based nanomaterials (Zavros and Merchant, 2022). The following sections will focus on responsive design strategies for metal-based nanomaterials tailored to the tumor microenvironment, analyzing their response mechanisms and therapeutic efficacy.

**FIGURE 8:**
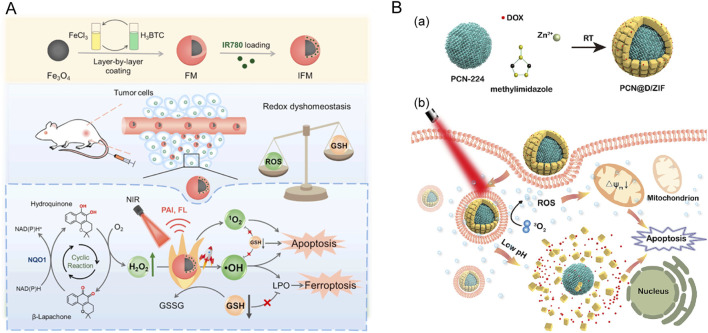
**(A)** Illustration of IFM for photo-enhanced upcycling H_2_O_2_ into highly cytotoxic ·OH for intense nanocatalytic tumor therapy. **(B)** (a) Schematic showing the synthesis process of PCN@D/ZIF NPs. (b) Schematic illustration of PCN@D/ZIF for PDT/Chemo-combined therapy (Liu et al., 2023c; Cun et al., 2022).

First, metal-based nanomaterials can function by responding to high H_2_O_2_ concentrations in the tumor microenvironment. For example, the IFM developed by Cun et al. (2022) utilized Fe_3_O_4_ NPs to harness high H_2_O_2_ concentrations in the tumor microenvironment. Through the Fenton reaction, these NPs decomposed H_2_O_2_ into highly cytotoxic ·OH radicals. The IR780 loaded in the encapsulation exhibited a photothermal effect under laser irradiation, accelerating the Fenton reaction. This synergistic effect significantly enhanced overall antitumor efficacy while reducing damage to normal tissues. The synthesis process and mechanism of action of IFM are illustrated in Figure 8A.

Additionally, there exists a class of metal-based nanomaterials responsive to the slightly acidic tumor microenvironment. For instance, the composite nanomedicine developed by Liu et al. (2023) employed a porphyrin MOF as its core, utilizing ZIF-8 to achieve the loading and assembly of the chemotherapeutic drug DOX. ZIF-8 underwent structural disintegration in the acidic tumor microenvironment, facilitating precise DOX release at the tumor site while exposing porphyrin-based MOF to synergistically exert therapeutic effects. This formulation exhibited outstanding pH-responsive release characteristics: minimal DOX leakage occured under neutral conditions, whereas the release rate reached 88.5% within 72 h at pH 5.0, effectively ensuring therapeutic targeting and safety. The synthesis process and mechanism of action of PCN@D/ZIF are illustrated in Figure 8B.

**FIGURE 9:**
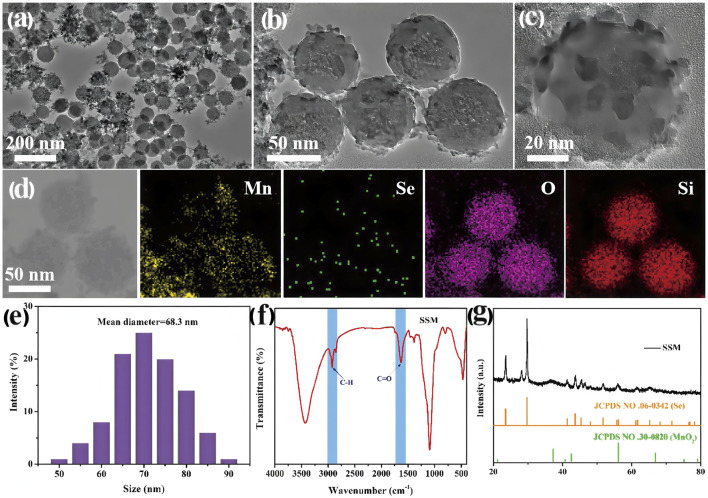
Characterization of SSM NCs. **(a)** Low-, **(b)** medium-, and **(c)** high-magnification TEM images of SSM NCs. **(d)** Element mapping images of Mn, Se, O and Si in SSM NCs. **(e)** Size distribution of SSM NCs measured by the DLS method. **(f)** FTIR spectra and **(g)** XRD pattern of SSM NCs (Wang et al., 2022b).

The design of modern nanomaterials increasingly focuses on integrating multi-responsive mechanisms to develop metal-based nanomaterials that activate exclusively within tumor tissues and exhibit multiple modes of action. This approach effectively overcomes the limitations of single-response strategies. For example, the BOD-Cu@G NPs designed by Jin et al. (2024) could simultaneously respond to high concentrations of GSH and H_2_O_2_ within tumor tissues. The high intracellular GSH concentration reduced Cu^2+^ to Cu^+^ within the BOD-Cu@G NPs, oxidizing GSH to oxidized glutathione (GSSG). This leaded to significant depletion of intracellular GSH, weakened the tumor cells’ antioxidant defense and further amplified the effects of ROS such as ^1^O_2_, thereby enhancing the efficacy of PT. Cu^+^ also underwent a Fenton-like reaction with high concentrations of H_2_O_2_ in tumor tissues, generating ·OH radicals to further achieve tumor killing. Similarly, the Se@SiO_2_@MnO_2_-ICG/DOX nanomaterial developed by Wang et al. (2022) exhibited triple responsiveness to tumor-specific conditions: high H_2_O_2_, mildly acidic pH, and elevated GSH levels (Figure 9). MnO_2_ dissolved in acidic conditions, releasing Mn^2+^ ions and DOX. GSH further reduced Mn^4+^ to Mn^2+^, which catalyzed a Fenton-like reaction with H_2_O_2_ to generate ·OH radicals, thereby achieving synergistic tumor therapy.

Overall, metal-based nanomaterials utilize responsive designs tailored to the specific characteristics of the tumor microenvironment to achieve precise carrier structure dissociation, controlled drug release, and synergistic enhancement of PT’s antitumor efficacy through catalytic reactions (Xiao and Yu, 2021). Integrated multi-stimulus response strategies are advancing the development of more efficient, low-side-effect precision cancer therapies. Future research should focus on improving the biocompatibility and controllable degradation properties of these nanomaterials. Additionally, comprehensive and systematic safety and efficacy evaluations in preclinical models are crucial to accelerate their translation into clinical applications.

## 5 Combination therapy based on metal-based nanomaterials for PT

The preceding section systematically outlined the developmental trajectory and numerous advantages of metal-based nanomaterials in PT. To further enhance tumor treatment efficacy, combined therapeutic strategies offer a highly promising avenue for optimization. This approach integrates the strengths of different therapeutic modalities, enabling a synergistic enhancement of antitumor effects. The following sections will focus on PDT and PTT, detailing synergistic treatment strategies that combine these modalities with other antitumor approaches.

### 5.1 Combined use of PT and chemotherapy

Chemotherapy is a widely used conventional approach for tumor treatment. However, traditional chemotherapeutic drugs inherently lack targeted selectivity and often cause systemic adverse effects, such as bone marrow suppression and nausea or vomiting during treatment. Additionally, some tumor cells readily develop drug resistance, increasing the risk of tumor recurrence after treatment (Kaushik et al., 2022). Metal-based nanomaterials exhibit exceptional tumor-targeting capabilities due to their unique design. They can induce tumor cell necrosis and apoptosis through photothermal effects, enabling rapid tumor cell destruction. Consequently, the combination of PDT, PTT, and chemotherapy holds great promise for significantly enhancing tumor-killing efficiency.

#### 5.1.1 Combination use of PDT and chemotherapy

**FIGURE 10:**
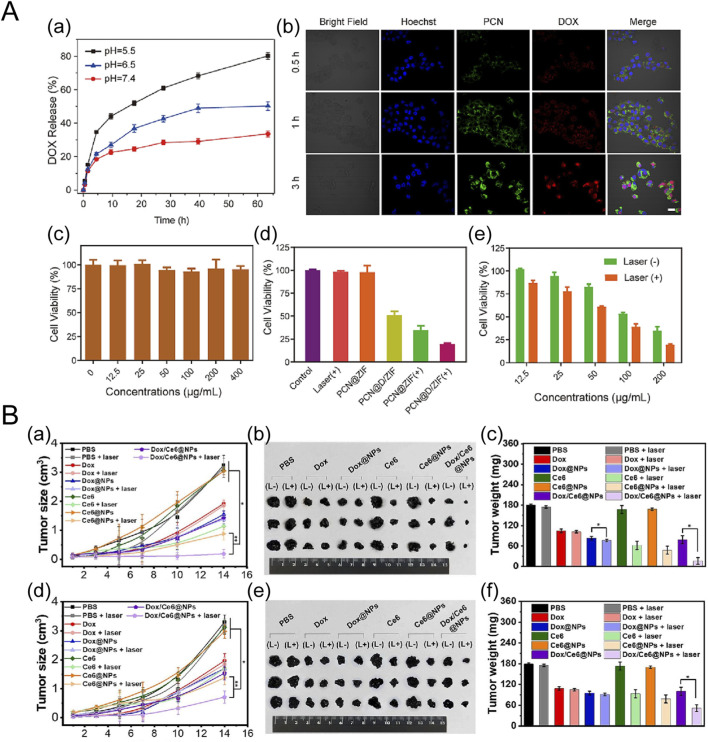
**(A)** (a) The DOX release profiles of PCN@D/ZIF (3.5 mg mL^-1^) at different pH values. (b) CLSM images of cells treated with PCN@D/ZIF (100 μg mL^-1^) for different times. (c) Viabilities of cells when treated with different concentrations of PCN@ZIF. (d) Viabilities of cells after different treatments (PCN@ZIF equivalent 200 μg mL^-1^). (e) Cell viabilities when treated with different concentrations of PCN@D/ZIF with or without light irradiation. **(B)** (a) Relative tumor volume over time after intratumoral injection. (b) Digital photos of tumors extracted at the end of therapy. (c) Changes in the weights of tumors for each tested group at the end of therapy. (d) Relative tumor volume over time after intravenous injection. (e) Digital photos of tumors extracted at the end of therapy. (f) Changes in the weights of tumors for each tested group at the end of therapy via intravenous injection. (L -) means without laser irradiation (laser -), (L +) means with laser irradiation (laser +) (Liu et al., 2023c; Yakubova et al., 2023).

PDT induces tumor cell apoptosis by generating ROS through the excitation of PSs. Building on this mechanism, chemotherapeutic drugs precisely target tumor tissues to exert their cytotoxic effects. Together, these approaches achieve a dual synergistic killing effect via ROS-mediated oxidative damage and the direct cytotoxicity of the chemotherapeutic agents. Some researchers have proposed developing metal-based nanomaterials loaded with chemotherapeutic drugs to combine PDT and chemotherapy for enhanced tumor cell eradication. As previously reported by Liu et al. (2023c), the PCN@D/ZIF nanomaterial designed loaded with DOX, generated substantial ROS under 655 nm laser irradiation and released DOX in response to the acidic tumor microenvironment, thereby enhancing selectivity and reducing chemotherapy side effects. This strategy combined the ROS generated by PDT with the dual-attack DNA damage and mitochondrial apoptosis signals induced by the chemotherapeutic agent DOX, significantly increasing tumor cell mortality (Figure 10A) while reducing the required drug dosage and minimizing effects on normal tissues. Additionally, Yakubova et al. (2023) designed a Ce6/Dox@CaCO_3_ system loaded with Ce6 and DOX based on calcium (Ca) carbonate, achieving equally satisfactory antitumor effects. They found that intratumoral injection further enhanced drug biodistribution and tumor accumulation efficiency, resulting in significantly higher tumor suppression rates compared to intravenous administration (94% vs. 71%) without notable toxicity to major organs (Figure 10B). Wang et al. (2020b) designed an RGD@am-ZnO@CuO@Au@DOX nanomaterial based on Zn, Cu, and Au to exert synergistic antitumor effects with DOX. Under NIR irradiation, Au NPs enhanced ROS production via LSPR amplification, thereby enabling PDT to kill tumor cells. Furthermore, NIR irradiation and the acidic tumor microenvironment synergistically promoted DOX release, enhancing the antitumor efficacy of chemotherapy.

Additionally, numerous studies have investigated the combination of PDT with chemotherapy drugs such as PTX and Methotrexate (MTX). The synergistic effects achieved through multiple pathways, dose optimization, overcoming drug resistance, and precise control via nano delivery enable this combination therapy to provide enhanced antitumor efficacy and improved safety compared to monotherapy. Consequently, it has become a prominent focus of research in contemporary cancer treatment.

#### 5.1.2 Combined use of PTT and chemotherapy

The synergistic mechanism of PTT combined with chemotherapy involves PTT directly killing tumor cells through heat generation by PTAs. Simultaneously, the elevated local temperature increases tumor cell membrane permeability and enhances tumor vascular permeability, thereby promoting the accumulation and intracellular uptake of chemotherapeutic drugs. Additionally, the thermal effect suppresses the activity of tumor cell resistance proteins, reversing chemotherapy resistance and ultimately achieving a synergistic enhancement of both thermal and drug-induced cytotoxicity.

Xing et al. (2023) prepared thermosensitive gold nanorod vesicles (UGRVs), which were loaded with DOX to form UGRV-DOX. Under 808 nm NIR irradiation, UGRV-DOX generated heat, enabling both PTT and promoting DOX release and intracellular uptake, thereby enhancing its cytotoxic efficacy. Results demonstrated that UGRV-DOX achieved a tumor inhibition rate of 99.3% under 808 nm laser irradiation, surpassing the 82.1% rate of the UGRV +808 nm laser group and the 51.2% rate of the DOX group, highlighting the significant advantage of combining PTT with chemotherapy. Additionally, PTT can reverse chemotherapy resistance in tumors. P-glycoprotein (P-gp) resistance refers to the enhanced ability of cancer cell membranes to efflux chemotherapy drugs, ultimately leading to tumor cell resistance to these agents. Khafaji et al. (2019) found that PEG-coated silica-encapsulated Fe_2_O_3_ NPs (PS-IONs) could improve P-gp resistance in certain cancer cells, thereby enhancing the tumor-killing effects of DOX and cisplatin (CDDP). With PS-IONs assistance, dual-drug chemotherapy significantly amplified cytotoxicity, reducing cell survival rates to 10.65% ± 0.9%.

This mechanism of drug release triggered by PTT-induced heat effectively enhances the selectivity of chemotherapy drugs toward tumor tissues while reducing toxic side effects on normal tissues. The combined therapy of chemotherapy and PTT can significantly enhance anti-tumor efficacy and reduce systemic toxicity through multiple mechanisms of action, including enhancing drug penetration through thermal effects, inhibiting tumor drug resistance, and activating the body’s immune system. It stands as one of the most promising “cocktail therapies” in contemporary nanomedicine.

### 5.2 Combined use of PT and radiotherapy

Radiotherapy is a well-established and widely used approach in tumor treatment, playing a crucial role in local tumor control. However, its antitumor efficacy is highly dependent on oxygen levels, as hypoxic tumor cells often exhibit significant resistance to radiotherapy. Furthermore, radiotherapy lacks selectivity between tumor tissue and surrounding healthy tissue, frequently causing severe systemic side effects (Yano et al., 2011). Furthermore, radiotherapy primarily achieves its cytotoxic effect by damaging tumor cell DNA through high-energy radiation or the ROS it induces. The robust DNA repair capabilities inherent to tumor cells significantly diminish sensitivity to radiotherapy (Dewey et al., 1995). The emergence of PT offers a novel and effective approach to overcoming these limitations of conventional radiotherapy.

#### 5.2.1 Combined use of PDT and radiotherapy

PDT using metal-based nanomaterials as PSs can both alleviate hypoxic conditions within the tumor microenvironment and promote efficient ROS generation, while also acting as radiosensitizers, reducing the toxic side effects of radiotherapy on normal tissues. Furthermore, PDT selectively damages tumor vascular endothelium and surrounding local tissues. When administered before or after radiotherapy, it decreases tumor volume and invasion depth, thereby expanding surgical indications or allowing for smaller resection margins to minimize surgical trauma.

For example, Shi et al. (2024) designed Bi_2_O_2_CO_3_:Yb/Er NSs based on Bi, ytterbium (Yb), and erbium (Er). Under excitation by a 980 nm laser, the Yb^3+^ doped in Bi_2_O_2_CO_3_:Yb/Er NSs absorbed energy and transferred it to Er^3+^, thereby generating red and green light. During tumor treatment, it was observed that under irradiation with 980 nm laser and 2 Gy X-rays, the Bi_2_O_2_CO_3_:Yb/Er NSs group (NSs + NIR + radiotherapy group) generated significantly more ROS compared to other treatment groups (e.g., NSs + NIR group and NSs + radiotherapy group), leading to marked damage to tumor cell DNA and mitochondria. Following irradiation with only 980 nm laser, tumor cell survival rate was 96.6%. After irradiation with only 2 Gy X-rays (radiotherapy group), tumor cell survival rate was 90.9%. This indicated that tumor killing by either 980 nm laser or 2 Gy X-rays alone was limited. Following the addition of Bi_2_O_2_CO_3_:Yb/Er NSs, tumor survival rates decreased to 51.0% and 48.0%, respectively. In the NSs + NIR + radiotherapy group, tumor survival rates further declined to 29.0%, indicating that the combination of PT and radiotherapy exhibited the strongest tumor-killing efficacy. After a 14-day treatment cycle, the NSs + NIR group exhibited noticeable thermal damage around the tumor site compared to the control group, while the radiotherapy group showed significant radiation damage. No obvious tissue damage was observed in the combined NSs + NIR + radiotherapy group. Further analysis of the tumor-adjacent tissues via H&E staining revealed that both the NSs + NIR group and the radiotherapy group exhibited clear signs of tissue damage, whereas the tissue morphology in the NSs + NIR + radiotherapy combination group remained largely normal. The results indicated that Bi_2_O_2_CO_3_:Yb/Er NSs could effectively suppress tumor growth while significantly mitigating the toxic side effects induced by high-energy X-ray or NIR irradiation.

#### 5.2.2 Combined use of PTT and radiotherapy

PTT using metal-based nanomaterials as PTAs generates localized thermal effects through NIR excitation, significantly increasing blood flow and oxygen levels in tumor tissues. This effectively reverses radiation resistance in hypoxic tumor cells. Additionally, the localized hyperthermia induced by PTT suppresses tumor cell DNA repair functions, elevating apoptosis rates. For radiotherapy- and heat-tolerant tumor cells, PTT disrupts their heat tolerance mechanisms (e.g., by inhibiting HSP90), restoring their sensitivity to radiation (Shahbazi et al., 2020). Notably, certain metal-based nanomaterials generate fluorescence or photoacoustic signals while mediating the PTT effect. This property enables real-time monitoring of temperature changes and dose distribution within the treatment area, providing a visual tool for tumor therapy monitoring.

Ma et al. (2022) designed NMOF545@Pt based on Pt NPs. Under 808 nm laser irradiation, the temperature of the NMOF545@Pt increased from approximately 25.4 °C to approximately 57.6 °C, enabling it to mediate PTT for killing tumor cells. Following additional 4 Gy X-ray irradiation, cell viability further decreased, with the killing effect increasing with higher NMOF545@Pt concentrations. *In vivo* mouse experiments demonstrated that NMOF545@Pt significantly enhanced the efficacy of PTT and radiotherapy, completely inhibiting tumor growth without noticeable side effects or organ damage. Additionally, NMOF545@Pt could enhance various imaging modalities including computed tomography (CT), magnetic resonance imaging (MRI), and photoacoustic imaging. This demonstrated its potential for real-time monitoring of temperature changes and dose distribution within the treatment area, providing a visual pathway for tumor therapy.

### 5.3 Combined use of PT and immunotherapy

Immunotherapy represents a promising approach to cancer treatment by activating the body’s immune system to suppress tumor growth. The most widely used strategy involves monoclonal antibodies that block immune checkpoints such as programmed cell death protein 1/programmed death ligand 1 (PD-1/PD-L1) and cytotoxic T-lymphocyte antigen-4 (CTLA-4), thereby restoring T-cell cytotoxic activity. The efficacy of this therapy depends on the extent of immune cell infiltration within the tumor microenvironment, making tumor blood flow and immune cell infiltration critical determinants of treatment response. PTT can enhance blood flow in tumor tissues to promote immune cell infiltration and induce tumor cell ICD, thereby releasing molecules such as calreticulin (CRT) and High Mobility Group Box 1 (HMGB1) that activate the immune system’s recognition and phagocytosis of tumor cells. Additionally, metal-based nanomaterials can downregulate PD-L1 expression on tumor cell surfaces, thereby enhancing the efficacy of immune checkpoint inhibitors.

Yang et al. (2025) designed AuNCs@PDA-Mn to promote dendritic cell (DC) maturation via PTT-induced ICD. DCs, as primary antigen-presenting cells, capture and process tumor-associated antigens in nearby draining lymph nodes while being activated by damage-associated molecular patterns to stimulate T cell activation. Subsequently, DCs migrate to tumor-draining lymph nodes and activate cytotoxic T lymphocytes (CD8^+^ T cells), helper T lymphocytes (CD4^+^ T cells), and natural killer cells (NK cells), ultimately secreting cytokines such as interleukin-6 (IL-6), interleukin-12 (IL-12), interferon-γ (IFN-γ), and tumor necrosis factor-α (TNF-α) to achieve tumor suppression. This synergistic effect enhances innate and adaptive antitumor immunity, achieving a systemic immune response. Furthermore, Wei et al. (2023) designed CuS/Z@M4T1 based on CuS NDs, wherein the coating of 4T1 tumor cell membranes endowed CuS/Z@M4T1 with homotargeted tumor cell recognition capability. Experimental results demonstrated that CuS/Z@M4T1 achieved cell death in the vast majority of tumor cells under 1064 nm laser irradiation. Regarding immune system activation, the PTT triggered by CuS/Z@M4T1 induced robust ICD and stimulated systemic antitumor immune responses. Furthermore, after CuS/Z@M4T1 converted “cold” tumors into “hot” tumors, the efficacy of PD-1 checkpoint inhibitors was significantly enhanced, exerting potent systemic immune suppression. This not only inhibited primary tumors in 4T1-bearing mice but also delayed metastatic lesions. *In vivo* experiments with 4T1-bearing mice demonstrated that the tumor volume in the CuS/Z@M4T1 + PD-1 checkpoint inhibitor group was significantly smaller than that in both the CuS/Z@M4T1 group and the PD-1 checkpoint inhibitor monotherapy group. This synergistic immune suppression effectively inhibits the growth of both primary and distant tumors, reducing the risk of tumor metastasis.

### 5.4 Combined use of PT and other therapies

In the field of tumor treatment, in addition to the three classic methods with widespread clinical applications, namely, chemotherapy, radiotherapy, and immunotherapy, there are several emerging treatment strategies that have not yet been widely adopted in clinical practice, such as sonodynamic therapy (SDT), starvation therapy (ST), and gas therapy (GT). Metal-based nanomaterials-mediated PT can also be combined with these emerging therapies to exert synergistic antitumor effects. (Kennedy, 2005; Cairns et al., 2011; Feng et al., 2018; Luo et al., 2025).

#### 5.4.1 Combined use of PT and SDT

Ultrasound forms the foundation of SDT for tumor treatment. As a mechanical wave with powerful penetrating capabilities, it can be utilized in cancer therapy (Kennedy, 2005). When focused within tissue, ultrasound generates intense cavitation effects, causing microbubbles to rupture and release high-energy free radicals. Under ultrasonic activation, the sonosensitizer further amplifies ROS production, creating a localized oxidative shockwave that kills tumor cells. However, the sonosensitizing activity of most current sound-activated agents remains relatively low, resulting in insufficient ROS generation. This limitation is especially pronounced in hypoxic tumor microenvironments, where effective oxidative killing is challenging (Hu et al., 2023). The combined application of PT and SDT mediated by metal-based nanomaterials, holds great promise for overcoming the current research bottleneck in SDT.

Based on this, Wen et al. (2022) synthesized H@Cu_9_S_8_@MnO_2_ multifunctional NPs, wherein Cu_9_S_8_ served as hollow spherical NPs for loading heme porphyrin PSs. Under 1064 nm laser irradiation, the MnO_2_ shell exhibited a 32.5% PCE, ensuring PTT efficacy. Furthermore, the MnO_2_ shell decomposed high concentrations of H_2_O_2_ in the tumor microenvironment into oxygen, improving the hypoxic conditions at the tumor site and providing a material basis for ROS generation. Following combined ultrasound and NIR irradiation, nearly all tumor cells were eliminated, confirming the potent tumor-killing efficacy of the SDT-PTT synergistic therapy. *In vivo* experiments yielded consistent results.

Overall, PT based on metal-based nanomaterials effectively overcomes the limitations of SDT. This therapy achieves comprehensive, efficient, precise, and low-toxic treatment effects on tumors through multiple mechanisms of action, including deep tissue penetration, improved oxygen supply, and dual cytotoxic effects achieved through ROS generation and heat production. Consequently, it represents one of the most promising multimodal anticancer strategies in modern nanomedicine.

#### 5.4.2 Combined use of PT and ST

The rapid growth of tumor cells requires an adequate supply of nutrients for maintenance. Glucose, the primary nutrient for tumor cell proliferation, is predominantly consumed through anaerobic glycolysis within these cells. Glucose oxidase (GOx), however, catalyzes the oxidation of glucose into H_2_O_2_ and gluconic acid in the presence of oxygen, thereby depleting glucose availability to cancer cells and inhibiting their proliferation. Based on this principle, researchers have proposed inhibiting tumor growth by disrupting glucose supply, as exemplified by ST (Cairns et al., 2011). However, ST can only inhibit tumor growth rather than directly kill tumor cells, suggesting that combining it with PT may provide a synergistic approach to tumor treatment.

Due to the frequent generation of toxic H_2_O_2_ and oxygen consumption during the cyclic operation of GOx, leading to severe systemic toxicity, Xu et al. (2022) developed a porous Cu(I) 1, 2, 4-triazole ([Cu(tz)]) carbon nanotube platform for GOx loading, ultimately yielding GOx@[Cu(tz)]. The non-porous Cu(tz) scaffold impeded the diffusion of glucose and oxygen, thereby limiting H_2_O_2_ production by GOx during the cycle and significantly reducing off-target toxicity. Compared to GOx alone, GOx@[Cu(tz)] exhibited a 188-fold increase in Michaelis constant (Km), retained over 86% of GOx activity, and demonstrated enhanced resistance to trypsin digestion. GOx@[Cu(tz)] was degraded by high concentrations of GSH in the tumor microenvironment, thereby consuming both GSH and glucose. Additionally, generated H_2_O_2_ can be converted by Cu(tz) into the more potent ·OH radical. Elevated H_2_O_2_ levels further enhance the PDT effect. Under 808 nm laser irradiation, this synergistic interaction ultimately induced 92.4% tumor growth inhibition, validating that GOx@ [Cu(tz)] exhibited high specificity and low systemic toxicity, suggesting its superiority in cancer therapy.

ST induces metabolic deprivation, placing tumor cells in an energy crisis state and reducing their tolerance to heat stress. PT further disrupts cellular structures through locally induced high temperatures or laser-generated ROS. The synergistic effect of these two approaches significantly overcomes the limitations of either PT or ST alone, offering a novel combined tumor therapy that opens new avenues for clinical antitumor treatment.

#### 5.4.3 Combined use of PT and GT

GT demonstrates unique antitumor potential through direct cytotoxicity, remodeling of the tumor microenvironment, cellular metabolic reprogramming, and synergistic effects with other therapeutic modalities. For instance, gas-induced cavitation can mediate irreversible cell necrosis. Nitric oxide (NO) induces cancer cell apoptosis and inhibits tumor angiogenesis via S-nitrosylation modification and regulation of the cGMP-PKG pathway, while also restoring local blood perfusion in hypoxic tumors. Both NO and carbon monoxide (CO) inhibit cytochrome c oxidase and other mitochondrial enzymes, thereby blocking tumor cell energy metabolism (Szabo, 2016; Yuan et al., 2025). However, GT has significant limitations, including rapid gas diffusion and a short half-life, which make it challenging to maintain precise and sustained local concentrations. Passive diffusion alone cannot ensure effective gas delivery to deep-seated tumors with poor blood supply, leading to inconsistent therapeutic outcomes (Zhou et al., 2023; Wang et al., 2024c). Consequently, researchers have proposed a combined GT-PT anticancer strategy based on metal-based nanomaterials. This approach employs laser irradiation to precisely mediate gas release at tumor sites, achieving accurate and sustained localized therapeutic gas concentrations. Additionally, the released gas enhances tumor cell sensitivity to ROS, thereby amplifying the PT’s tumor cell-killing effects (Wan et al., 2018).

Wu et al. (2021) prepared Cu-loaded cobalt-Cu-Fe NSs (CCFS NSs) using CoCuFe layered double hydroxide (LDH) NSs as the raw material. CCFS NSs exhibited strong light absorption at 808 nm, with higher PCE in acidic tumor microenvironment (72.0% at pH 7.4% vs. 81.0% at pH 5.4). Furthermore, high concentrations of H_2_O_2_ in the tumor microenvironment triggered an explosive release of NO, achieving precise and sustained local gas concentrations necessary for GT tumor treatment. *In vitro* and *in vivo* experiments demonstrated that under 808 nm laser irradiation, tumor cells treated with CCFS NSs exhibited an apoptosis rate of 91.8%, approaching complete elimination, fully demonstrating the efficacy of the combined GT and PT treatment strategy.

The combined use of GT and PT achieves enhanced antitumor efficacy through thermally triggered gas release, synergistically increased ROS generation, and modulation of the tumor microenvironment, emerging as a promising approach for multimodal cancer therapy using nanomaterials (Yan et al., 2024).

In summary, although these emerging cancer therapies have demonstrated considerable therapeutic potential, they still face numerous limitations. These treatments are far from clinical application and urgently require further validation and investigation.

## 6 Advances in preclinical research and prospects for clinical translation

Looking back at the development journey, PT based on metal-based nanomaterials has advanced rapidly in the preclinical research phase. Leveraging the exceptional properties of metal-based nanomaterials, PT has achieved significant progress in the field of preclinical tumor therapy research. Examples include the previously mentioned Au@mSiO_2_-ICG nano-dumbbells, PCN@D/ZIF, and BOD-Cu@G, all of which represent key components driving PT toward clinical application. The following discussion aims to summarize the achievements of PT based on metal-based nanomaterials in preclinical tumor treatment research and to explore the challenges these systems encounter during clinical translation.

### 6.1 Advances in preclinical research

As mentioned above, PT relies on light irradiation to exert its effects. With the continuous development of PSs and PTAs, particularly advances in metal-based nanomaterials, preclinical research on PT for tumor treatment has become increasingly sophisticated. Researchers can modify the size and shape of metal-based nanomaterials, optimize their responsive design, and perform surface functionalization to achieve diverse effects. These include red or blue shifts in absorption peaks, enhanced PCE, increased ROS generation capacity, and improved targeting capabilities. Leveraging these properties, metal-based nanomaterials have demonstrated exceptional tumor-killing efficacy in preclinical studies across multiple malignant tumor types.

**FIGURE 11:**
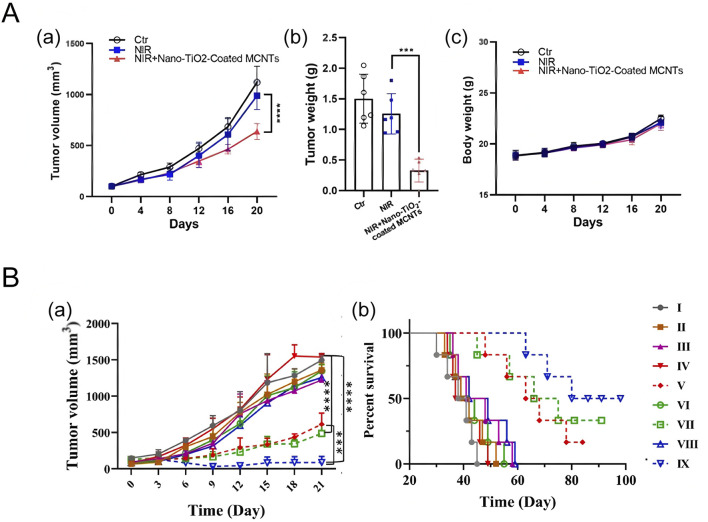
**(A)** Nano-TiO_2_-coated MCNTs enhance the tumor-killing ability of NIR laser irradiation in tumor xenografts. (a) Tumor volume was recorded every four days until the day 20th. (b) At the end of the animal study, mice were sacrificed, and tumors were excised for weighing. (c) Body weight was also recorded every 4 days until the day 20th. **(B)** *In vivo* anti-tumor effects of IR780/Ce@EGCG/APT. (a) Growth profiles of tumors after different treatments. (b) Survival curves of 4T1 tumor-bearing mice during treatments. I: NSs, II: Ce@EGCG, III: Ce@EGCG/APT, IV: IR780, V: IR780 + (L), VI: IR780/Ce@EGCG, VII: IR780/Ce@EGCG + (L), VIII: IR780/Ce@EGCG/APT, IX: IR780/Ce@EGCG/APT + (L) (Mu et al., 2024; Xie et al., 2024).

Globally, colorectal malignancies rank as the third most common cancer type and the second leading cause of cancer-related deaths (Bray et al., 2024). As mentioned earlier, Xie et al. (2024) prepared nano-TiO_2_-coated MCNTs. In the culture medium of colon cancer cells, when the concentration of nano-TiO_2_-coated MCNTs reached 200 μg mL^-1^ and the cells were irradiated with an 808 nm laser at 1.5 W cm^-2^ for 5 min, followed by 24 h of incubation, cell viability decreased to 30%. Researchers implanted colon cancer cells into mice. After 20 days of cultivation, the experimental group treated with 200 μg mL^-1^ nano-TiO_2_-coated MCNTs and irradiated with an 808 nm laser at 1.5 W cm^-2^ for 5 min exhibited significantly reduced tumor weight and volume compared to the untreated subgroup (Figure 11A).

Within the realm of malignant breast tumors, triple-negative breast cancer stands as one of the most aggressive forms, exhibiting resistance to numerous conventional anticancer therapies (Li et al. 2022b). As mentioned earlier, Mu et al. (2024) designed IR780/Ce@EGCG/APT. In the culture medium of triple-negative breast cancer cells, when IR780/Ce@EGCG/APT was set at a concentration of 3 μg mL^-1^ and irradiated with an 808 nm laser at 1.0 W cm^-2^ for 5 min, cell viability decreased to 20% after 20 h of incubation. After implanting triple-negative breast cancer 4T1 cells into mice and housing them for 10 days, the experimental group treated with IR780/Ce@EGCG/APT and 808 nm laser exhibited significantly smaller tumor volumes compared to other subgroups (Figure 11B). Furthermore, the survival rate of mice in the experimental group showed a marked improvement during prolonged housing.

Lung cancer is one of the most common malignant tumors in most countries and remains the leading cause of cancer-related deaths worldwide (Kratzer et al., 2024). As mentioned earlier, Yet et al. (Ye et al., 2014) designed Au@Ag NSPs. After adding 2.2 × 10^10^ mL^-1^ Au@Ag NSPs to the culture medium of lung adenocarcinoma cells and irradiating them with a 980 nm laser for 5 min, microscopic observation after 6 h of incubation revealed that the majority of lung adenocarcinoma cells had died. Subsequently, researchers implanted lung adenocarcinoma cells into mice and maintained them for 10 days. Results showed that the experimental group treated with Au@Ag NSPs and exposed to 980 nm laser irradiation for 5 min exhibited significantly higher tumor necrosis levels compared to other subgroups.

Although PT based on metal-based nanomaterials has demonstrated remarkable therapeutic efficacy and broad application potential in preclinical studies of various malignant tumors, certain limitations hinder its practical application. These include suboptimal biocompatibility, limited tissue penetration, and relatively short circulation half-lives. While most preclinical studies have confirmed the biocompatibility of these nanomaterials, research on their effects on human cells and the body as a whole remains limited during the transition from preclinical to clinical trial phases. Therefore, extensive and in-depth investigations are still necessary.

### 6.2 Prospects for clinical translation

The long-term biocompatibility and *in vivo* metabolic mechanisms of these nanomaterials remain incompletely understood. Although the volume of related research continues to grow, the number of approved nanomedicines has not increased significantly. Nevertheless, as research advances, a series of challenges hindering the clinical application of PT based on metal-based nanomaterials are gradually being addressed.

First, the biocompatibility of metal-based nanomaterials is a critical factor determining their effective functionality. The PC mentioned earlier is one of the key issues affecting the biocompatibility of these nanomaterials. The formation of the PC causes metal-based nanomaterials to be more readily recognized and cleared by macrophages, thereby shortening the half-life of NPs (Xiao et al., 2022). Furthermore, PC also reduces the stability of metal-based nanomaterials. For example, Cao et al. (2019) found that the PC formed around orally delivered NPs may significantly affect their stability in the gastrointestinal tract. Furthermore, the PC may also affect the ability of metal-based nanoscale systems to target tumor cells. For instance, Nemati et al. (2021) prepared a PC coating using plasma from breast cancer patients and demonstrated that the PC significantly suppressed the targeting properties of FA-modified chitosan NPs, thereby hindering their uptake by tumor cells. Adding PEG to the surface of NPs reduces the formation of PC and improves the half-life of the NPs in the body. Therefore, to mitigate the impact of PC on metal-based nanomaterials, researchers have proposed PEGylation as a method to decrease PC formation.

However, PEGylation of drugs is not without its drawbacks. Ishida et al. (2005) discovered that following the first intravenous injection, the body produced specific antibodies against PEG. This antibody production significantly shortened the circulation time of subsequent liposome administrations, thereby reducing therapeutic efficacy. In light of this, another method to reduce PC formation has emerged: treating drugs with poly-L-glutamic acid (PGA). PGA is a non-toxic, non-immunogenic, and biodegradable polyamino acid. Research by Karabasz et al. (2019) indicated that PGA-coated nanocapsules (NC-PGA) exhibited similar pharmacokinetic and biodistribution characteristics to PEG-coated nanocapsules (NC-PEG). However, compared to repeated administration of NC-PGA, NC-PEG nanocapsules significantly elevated serum cytokine levels, thereby increasing PC production. This indicates that PGA is a more biocompatible invisible polymer, holding positive implications for advancing metal-based nanomaterials-driven PT toward clinical application.

Secondly, the targeting capability of metal-based nanomaterials is equally critical for the efficacy and safety of PT. Developing effective targeting mechanisms is essential to enhance therapeutic specificity, with surface functionalization using tumor-specific ligands being a common approach. As mentioned earlier, given the high expression of FRs in tumor tissues, conjugating FA to the nanomaterials can significantly improve targeting of tumor tissues (Moonshi et al., 2023). Additionally, some scholars have proposed that responsive designs for metal-based nanomaterials can be developed by leveraging the unique characteristics of the tumor microenvironment, such as its mildly acidic pH, high concentrations of H_2_O_2_, and elevated levels of GSH. This strategy aims to create metal-based nanomaterials that demonstrate therapeutic efficacy exclusively within tumor tissues. For example, the CCFS NSs designed by Wu et al. (2021) achieved higher PCE by leveraging the mildly acidic conditions (pH 5.4) in the tumor microenvironment, thereby preventing CCFS NSs from accumulating in and damaging normal tissues. Xu et al. (2022) designed GOx@[Cu(tz)], wherein the non-porous Cu(tz) scaffold impeded diffusion of glucose and oxygen while responding to high GSH concentrations in the tumor microenvironment, significantly reducing GOx’s off-target toxicity. Wang et al. (2022b) designed Se@SiO_2_@MnO_2_-ICG/DOX, which utilized high concentrations of H_2_O_2_ in the tumor microenvironment to promote its decomposition, thereby releasing drugs such as Mn and DOX to exert antitumor effects. This approach achieved protection of normal tissues while inhibiting tumor cells.

Non-precious metal-based nanomaterials are widely used metallic materials in practice. After being engineered into nanoscale forms, they can effectively address biosafety concerns. For example, Fe-based nanomaterials can degrade into ferrous ions under acidic conditions in TME, and ultimately be converted into iron ions that can be cleared by the body through biological metabolic pathways, reducing long-term retention toxicity. Cu-based nanomaterials gradually oxidize and release copper ions in the body. Excess copper accumulation can induce lipid peroxidation and oxidative stress damage, mainly accumulating in the liver and being excreted through bile, with some being excreted through urine. However, the specific renal clearance threshold is not yet clear. Long-term exposure to Co-based nanomaterials can lead to accumulation in tissues such as the liver, kidneys, and heart, which are easily excreted through feces and urine through a three-stage kinetic process. These properties theoretically guarantee their biological safety and facilitate their further clinical translation.

Metal-based nanomaterials are progressing toward clinical applications due to their multifunctionality and exceptional diagnostic and therapeutic potential. To enable widespread medical translation, it is essential to overcome challenges related to efficacy and biosafety, while scalable production and adherence to regulatory standards remain equally critical. Currently, numerous technical limitations hinder the batch-scale, reproducible synthesis of high-purity materials. Additionally, high costs and complex manufacturing processes present significant obstacles to clinical implementation (Yu et al., 2025). Currently, the entire field lacks unified standards for quality control, toxicity assessment, and risk management. Establishing regulatory oversight for PT-related products is a key factor in ensuring their successful transition to clinical use. Achieving this goal requires coordinated efforts and collaboration among multiple departments (Cai et al., 2025). For example, in 2022, the U.S. Food and Drug Administration (FDA) issued guidelines for drugs containing nanomaterials, requiring that the release of metal ions be less than 0.1 ppm (in simulated biological fluid for 30 days), conduct chronic toxicology studies (on two relevant species) for 6–12 months, and complete a comprehensive analysis of biodistribution and excretion. Secondly, the European Medicines Agency (EMA) has issued relevant guidelines for Advanced Therapeutic Medicinal Products (ATMPs), which accept mutagenicity studies in transgenic rodents (OECD 488), and adjust safety assessment standards according to specific circumstances, requiring specialized toxicokinetic studies. Overall, regulatory authorities emphasize material-specific assessments, metal ion release control, long-term toxicity studies, and complete biodistribution/clearance analysis for inorganic nanomaterials. Relevant guidelines are still being continuously updated and improved.

### 6.3 Future development trends

Beyond addressing challenges in clinical treatment, clarifying the future development trajectory of metal-based nanomaterials remains a critical research focus. Currently, intelligent and responsive metal-based nanomaterials capable of functioning in response to external stimuli have demonstrated promising results in preclinical studies. Research and development of metal-based nanomaterials with self-healing and self-assembly capabilities have also formally begun. These systems utilize metal-polymer or MOF frameworks to achieve structural reconfiguration upon environmental sensing. The goal is to enable “single-dose delivery with multiple-dose control”, working in conjunction with AI-driven diagnostic platforms to achieve precise regulation of drug release and catalytic activity (Wojnicki et al., 2024; Chen et al., 2025). For example, the AI-EDISON robotic system at the University of Glasgow automatically explores high-dimensional synthesis parameter space through Bayesian optimization algorithms, enabling autonomous discovery and optimization of nanoparticle synthesis conditions. It can regulate key parameters such as temperature, time, and precursor ratio in real-time (Jiang et al., 2022). A collaboration between Cardiff University and AstraZeneca has proven that AI can efficiently optimize various synthetic parameters. This method helps develop better nanoparticles compared with early versions. Through computational learning, researchers can fabricate high-performance nanoparticles. These carriers possess higher stability and drug-loading capacity, enabling precise drug accumulation at tumor sites and improving therapeutic effects (Serrano et al., 2024). AI has demonstrated outstanding performance in optimizing the stability and drug-loading capacity of nanocarriers, providing crucial technical support for precision cancer treatment.

On the other hand, multifunctional metal-based nanomaterials have demonstrated rapid development in preclinical research. For instance, the AgIONPs designed by Moonshi et al. (2023) not only possessed PTT functionality but also MRI capability, proving effective in both tumor treatment and diagnosis. Similarly, the NMOF545@Pt system developed by Ma et al. (2022) combined tumor therapy with enhanced imaging capabilities across CT, MRI, and photoacoustic modalities. These multifunctional metal-based nanomaterials aim to achieve synergistic operation between diagnostic functions and PDT/PTT antitumor therapies (Wen et al., 2019; Barik and Konkimalla, 2025; Ma et al., 2025). To further enhance the safety of metal-based nanomaterials, synthesizing low-toxicity and biodegradable variants will become the primary focus in the future. Green synthesis methods offer a promising approach to achieve this goal. Techniques such as plant- or microbe-assisted reduction and solvent-free processes aim to balance the functionality and safety of metal-based nanomaterials, enabling them to self-degrade after fulfilling their intended purpose, thereby minimizing adverse effects (Manivasagan et al., 2015; Li et al., 2018; Liu et al. (2024b); Wang et al., 2026).

To overcome limitations in production capacity, researchers have begun employing micro-nano 3D printing and *in situ* deposition techniques to synthesize metal-based nanomaterials. By leveraging continuous flow synthesis processes, they aim to achieve mass production while ensuring dimensional and morphological consistency (Adir et al., 2020; Chauhan et al., 2023). Furthermore, with the continuous advancement of artificial intelligence technology, intelligent composite or self-sensing metal-based nanomaterials have emerged as a new development trend. Specifically, such systems incorporate micro sensors or actuators, such as strain gauges and temperature sensors. The goal is to create metal-based nanomaterials capable of self-monitoring and self-regulating under complex environmental conditions (Lee and Lo, 2024; Younis et al., 2021; Yu et al., 2025).

Looking ahead, metal-based nanomaterials are expected to evolve from single-function systems into intelligent, multifunctional, and low-toxicity platforms. The technical pathways to achieve this transformation include responsive design, functional integration, green synthesis, and AI-assisted approaches. Once successfully developed, these metal-based nanomaterials will undoubtedly exhibit enhanced efficacy and improved safety in PT and combination therapy applications.

## 7 Conclusion

Over the past few decades, PT has demonstrated remarkable therapeutic efficacy and broad clinical prospects, particularly in oncology. However, to surmount the intrinsic limitations of conventional PSs and PTAs, metal-based nanomaterials have emerged as a dominant paradigm due to their superior optical and thermal physicochemical properties. As highlighted in this review, precious metal nanomaterials (e.g., Au, Ag) have secured a prominent position in PT owing to their tunable LSPR and exceptional PCE. Simultaneously, non-precious metal nanomaterials (e.g., Fe, Mn) have become increasingly indispensable due to their cost-effectiveness, abundance, and unique intrinsic catalytic activities. Collectively, these advancements establish a robust material foundation for achieving highly efficient, spatially controllable PT.

Despite this progress, the transition from preclinical success to clinical utility is hindered by challenges such as potential cytotoxicity and suboptimal tumor accumulation. Consequently, the rational design of physicochemical properties and responsiveness is paramount. We have underscored that precise control over size and morphology is fundamental for optimizing optical absorption windows, while surface functionalization and stimulus-responsive engineering are critical for enhancing biocompatibility and conquering biological barriers. Through these multifaceted optimization strategies, metal-based nanomaterials have achieved significantly improved safety profiles and therapeutic indices in preclinical models, laying the groundwork for future clinical translation.

Furthermore, moving beyond monotherapy to synergistic combination modalities represents a critical evolution in cancer treatment. There is a growing trend toward integrating metal-based PT with established modalities (e.g., chemotherapy, radiotherapy, and immunotherapy) to combat tumor heterogeneity and multidrug resistance. Moreover, the incorporation of emerging techniques like SDT and ST offers novel perspectives for maximizing therapeutic outcomes. Although these integrated approaches require further validation, they provide powerful strategies to elicit systemic antitumor immunity and eradicate metastatic lesions.

Looking ahead, while metal-based nanomaterials have demonstrated remarkable potential in preclinical PT through high therapeutic efficiency and functional versatility, significant hurdles remain in transitioning these findings from bench to bedside. These translational challenges encompass optimizing the therapeutic index, ensuring long-term biosafety, achieving reproducible large-scale manufacturing, and navigating complex regulatory landscapes. We firmly believe that through concerted interdisciplinary efforts, metal-based PT will achieve pivotal breakthroughs in clinical translation, ultimately providing safe and effective treatment paradigms for cancer patients.
